# Meat, dairy and plant proteins alter bacterial composition of rat gut bacteria

**DOI:** 10.1038/srep15220

**Published:** 2015-10-14

**Authors:** Yingying Zhu, Xisha Lin, Fan Zhao, Xuebin Shi, He Li, Yingqiu Li, Weiyun Zhu, Xinglian Xu, Chunbao Lu, Guanghong Zhou

**Affiliations:** 1 Key Laboratory of Meat Processing and Quality Control, MOE; Key Laboratory of Animal Products Processing, MOA; Jiang Synergetic Innovation Center of Meat Processing and Quality Control; Synergetic Innovation Center of Food Safety and Nutrition; Nanjing Agricultural University; Nanjing 210095, P.R. China; 2 Gastrointestinal Microbiology Joint Research Center; Laboratory of Gastrointestinal Microbiology; Nanjing Agricultural University; Nanjing 210095, P.R. China

## Abstract

Long-term consumption of red meat has been considered a potential risk to gut health, but this is based on clinic investigations, excessive intake of fat, heme and some injurious compounds formed during cooking or additions to processed meat products. Whether intake of red meat protein affects gut bacteria and the health of the host remains unclear. In this work, we compared the composition of gut bacteria in the caecum, by sequencing the V4-V5 region of 16S ribosomal RNA gene, obtained from rats fed with proteins from red meat (beef and pork), white meat (chicken and fish) and other sources (casein and soy). The results showed significant differences in profiles of gut bacteria between the six diet groups. Rats fed with meat proteins had a similar overall structure of caecal bacterial communities separated from those fed non-meat proteins. The beneficial genus *Lactobacillus* was higher in the white meat than in the red meat or non-meat protein groups. Also, rats fed with meat proteins and casein had significantly lower levels of lipopolysaccharide-binding proteins, suggesting that the intake of meat proteins may maintain a more balanced composition of gut bacteria, thereby reducing the antigen load and inflammatory response in the host.

Although some epidemiological evidence suggests that the consumption of meat, especially red meat (beef and pork), might lead to higher incidence of cardiovascular disease (CVD) and colorectal cancer (CRC)^1^,^2^,^3^,^4^, meat remains an important component of the human diet since it contains high quality proteins, amino acids, fatty acids, minerals, and vitamins^5^. Studies examining meat intake in relation to human health are mainly focused on the effect of excessive intakes of red meat on health. Few data are available on the effect of type of dietary proteins on human health. One study has reported that substituting of red meat proteins with fish, poultry, or soy proteins would decrease the risk of mortality resulting from CVD and CRC^6^. This study suggested that red meat protein might not be as healthy compared with the other sources of proteins. However, the underlying reason for this is not clear.

Gut bacteria in the caecum and colon have been recently recognized as an important player between food and the host^7^. Gut bacteria may affect human physiology and nutrition, such as food digestion, immune cell development and homeostasis, fat metabolism regulation, angiogenesis promotion, enteric nerve regulation, and epithelial homeostasis^8^,^9^,^10^. The composition of gut bacteria may be affected by host’s physiology, pathology, environment, immune system and lifestyle, and diet is a very important factor^7^. Foods are digested and absorbed in the stomach and small intestine, but a substantial quantity of food may enter into large intestine and alter the diversity of gut bacteria^11^,^12^. For example, intake of a high fat diet has been shown to decrease the relative abundance of *Bacteroidetes* and *Bifidobacteria* in rat feces, but to increase the relative abundance of *Firmicutes* and *Proteobacteria*^13^. In various studies, the predominant gut bacteria in vegetarians was found to be *Clostridium coccoides, Faecalibacterium Prausnitzii* and *Clostridium ramosum*, but *Clostridium cluster XIVa* was the characteristic bacterium for omnivores^14^,^15^,^16^.

More recently, an excessive intake of red meat has been shown to cause gut health problems possibly due to heme, N-nitroso compounds, polycyclic aromatic hydrocarbons and hetero-cyclic amines in cooked meat products^17^, or possibly due to the presence of glycan^18^. However, red meat is still an important source for protein. In particular, our previous study showed that short-term intake of meat proteins at the recommended level would lead to significantly greater diversity of gut bacteria in rat feces compared with those fed with soy protein. The effects of long-term intake of meat proteins on the diversity of gut bacteria and host health remain unknown.

The objective of this study was to examine how dietary proteins (at recommended intake level) from red meat (beef and pork), white meat (chicken and fish), dairy (casein) and plant (soy) proteins affect the diversity of caecal bacteria and serum lipopolysaccharide-binding protein (LBP) to evaluate the antigen load from gut bacteria to the rat. The aims were to determine if dietary protein was a critical regulatory factor for caecal bacteria, and if the intake of meat proteins at recommended level reduced the antigen load from gut bacteria to the host.

## Results

### Richness and diversity of gut bacteria

Under the 16S rRNA sequencing platform, a total of 1,997,831 usable raw reads were obtained from all 64 samples with an average of 31,216 ± 4,706 reads per sample (see Supplementary Fig. S1a online). Two samples were not included because one rat from the casein group and one from soy protein group died during the course of feeding. Operational taxonomic units (OTU) were delineated at the 97% similarity level. The total number of OTU was 22,623, with an average of 353 ± 51 per sample. Although no rarefaction curve reached a plateau (see Supplementary Fig. S1b online), the Shannon-Wiener diversity index estimates were stable for all 64 samples (see Supplementary Fig. S1c online). This suggests that new phylotypes would be expected if additional sequencing was performed, but most species have been captured. The Good’s coverage index (99.75% ± 0.04%) showed that this sequencing method can characterize the true composition of gut bacteria. There was no significant difference (p > 0.05) between any two groups in ACE, Chao, Shannon index, Simpson index, and Good’s coverage index (see Supplementary Table S1 online).

### Overall structure of gut bacteria

**Figure 1:**
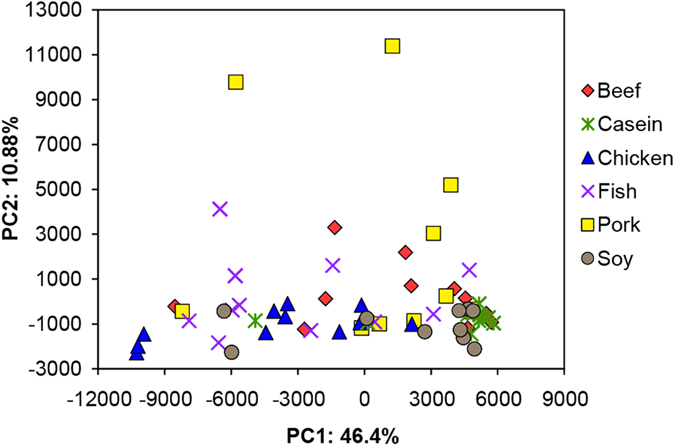
Principal component analysis of gut bacteria at the OTU level. The numbers of animals for beef, casein, chicken, fish, pork and soy protein groups are 11, 10, 11, 11, 11, and 10, respectively. Red, olive green, blue, purple, yellow and grey color represent beef, casein, chicken, fish, pork, and soy protein groups, respectively. Each point represents one animal.

Multivariate analyses were performed to compare the overall composition of caecal bacteria among all samples at the OTU level. Principal component analysis (PCA) revealed a substantial inter- and intra- group variation of gut bacteria as a response to dietary proteins (Fig. 1). The first two components accounted for 57.28% of the total variation. PC1 interpreted both inter-group and intra-group variations, while PC2 mainly explained the intra-group variation from rats fed with pork protein. The composition of gut bacteria in rats fed with soy protein and casein was similar with relatively small intra-group variation, but rats fed with chicken and fish proteins showed a great intra-group variation. This indicates that the composition of gut bacteria exhibits a diverse response to meat proteins.

**Figure 2:**
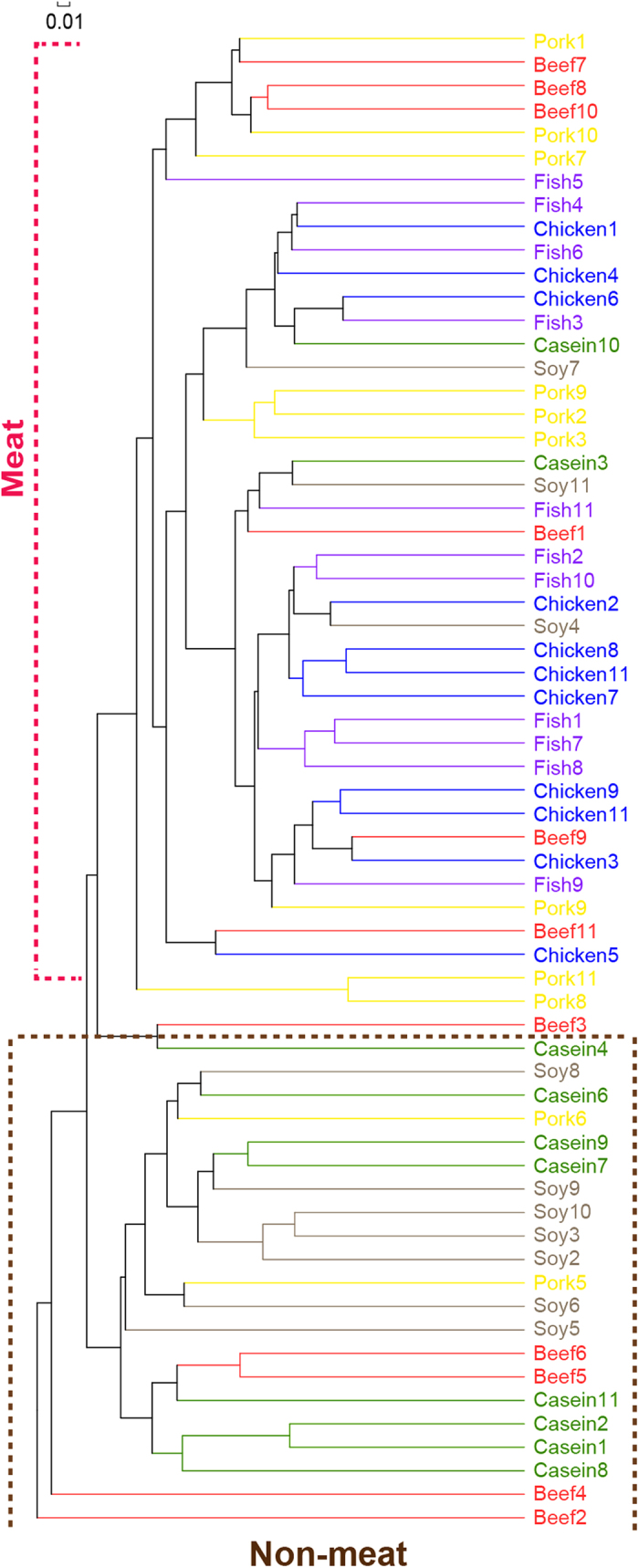
Hierarchical clustering of gut bacteria at the OTU level. The numbers of animals for beef, casein, chicken, fish, pork and soy protein groups are 11, 10, 11, 11, 11 and 10 respectively. Red, olive green, blue, purple, yellow and grey color represent beef, casein, chicken, fish, pork, and soy protein groups, respectively. Each line represents one animal.

Bray-Curtis clustering analysis of gut bacteria at the OTU level indicated that all samples can be clustered into two subgroups, i.e., non-meat (casein and soy proteins, the bottom cluster, Fig. 2) and meat (fish, chicken, beef and pork proteins, the top cluster). This is in agreement with the PCA results.

### Composition of gut bacteria

**Figure 3:**
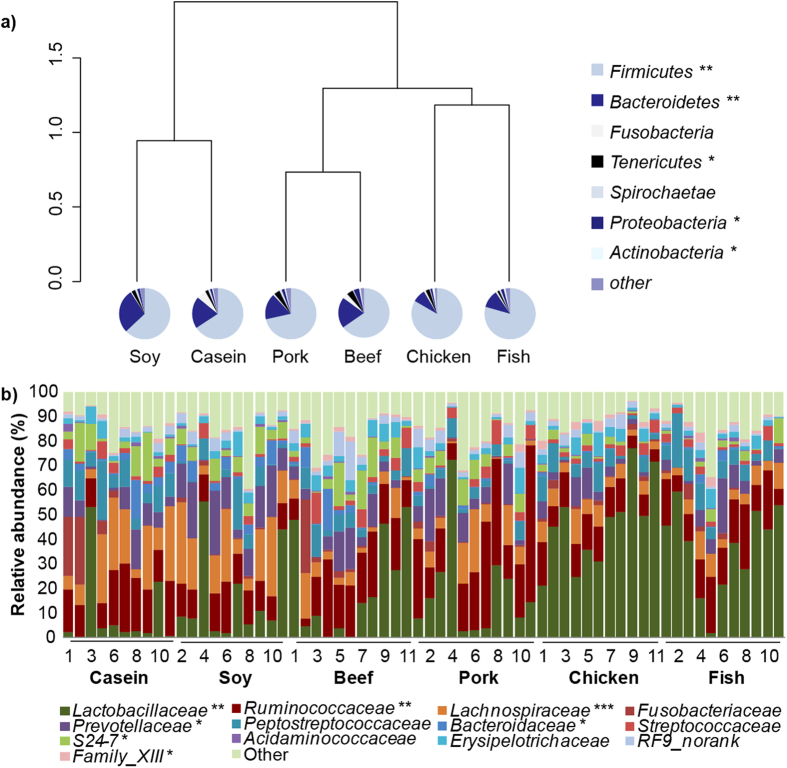
The composition of gut bacteria in caecum at the phylum and family levels. Relative abundance of the different phyla in response to different dietary proteins (average percentage). Bray-Curtis similarity clustering analysis of caecal bacteria at the phylum level showed that six groups can be clustered into three classes: white meat protein class (chicken and fish protein groups), red meat protein class (beef and pork protein groups) and non-meat protein class (casein and soy protein group). Taxon-based analysis at the family level. Each column represents one animal. Note: The numbers of animals for beef, casein, chicken, fish, pork and soy protein groups are 11, 10, 11, 11, 11 and 10 respectively. Data were analyzed by one-way analysis of variance and means were compared by Duncan’s multiple comparison.

At the phylum level, the six groups could be divided into three clusters: 1) rats fed with casein and soy protein; 2) rats fed with pork and beef proteins; 3) rats fed with chicken and fish proteins (Fig. 3a). *Firmicutes* and *Bacteroidetes* were the two most predominant phyla in all six groups, contributing 41.17% ~ 97.80% and 0.21% ~ 47.62% of the total OTU, respectively. Rats fed with chicken and fish proteins had higher *Firmicutes* but lower *Bacteroidetes* than those fed with other proteins. Compared with the other five groups, rats fed with soy protein had a higher abundance of *Bacteroidetes*, whereas rats fed with chicken protein had greater abundance of *Actinobacteria* and rats fed beef protein had greater abundance of *Proteobacteria* (see Supplementary Fig. S2 online).

At the family level (Fig. 3b), rats fed with soy protein and casein has a similar profile of gut bacteria, which was characteristic of *Lachnospiraceae* (average: 17% and 18%, respectively). However, *Ruminococcaceae* (average: 18% and 27%, respectively) and *Lactobacillaceae* (average: 20% and 19%, respectively) were the characteristic bacteria in rats fed with beef and pork proteins, and *Lactobacillaceae* (average: 46% and 36%, respectively) was characteristic for those fed with chicken and fish proteins.

### Linear discriminant analysis of gut bacteria

The above results indicated that all 64 samples could be simply grouped into three classes of red meat proteins, white meat proteins and non-meat proteins. Each class had a similar composition of gut bacteria. To identify specific bacteria that are characteristic for the three classes, linear discriminant analysis effect size (LEfSe) was applied.

**Figure 4:**
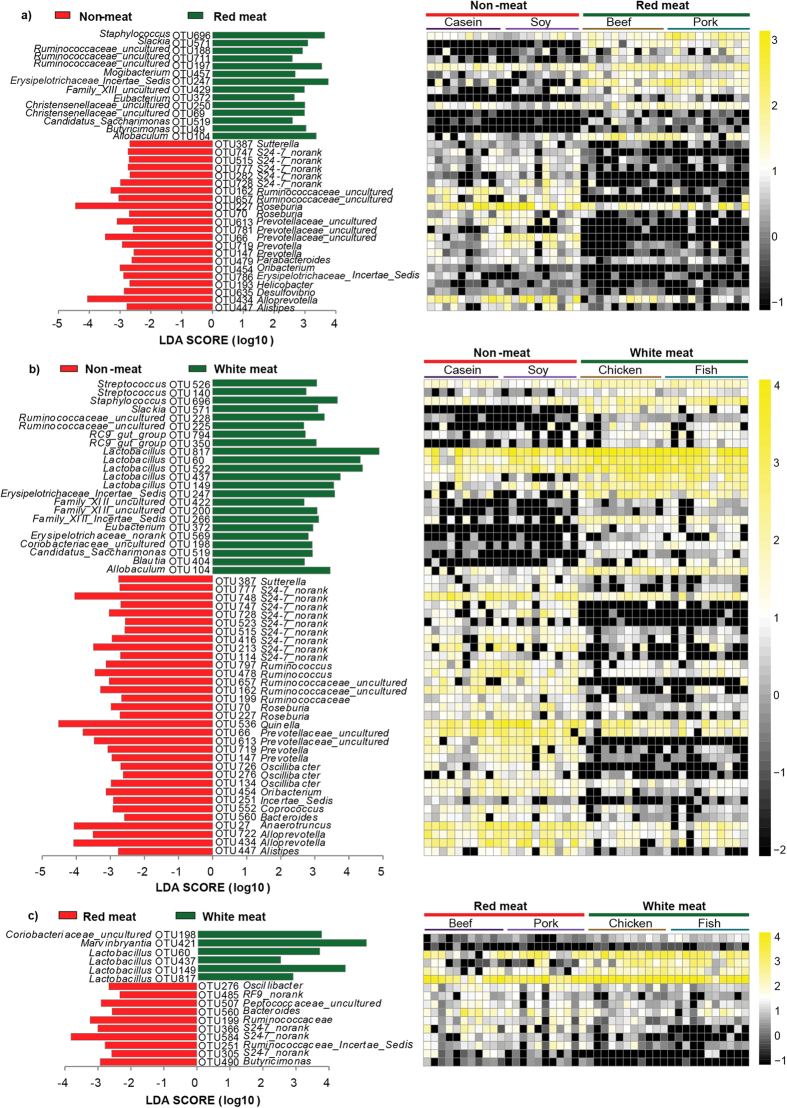
Comparisons of caecal bacteria using LefSe. (**a**) between non-meat protein class and red meat protein class; (**b**) between non-meat protein class and white meat protein class; (**c**) between red meat protein class and white meat protein class with significant diversity (p < 0.001, one-way ANOVA) Note: The numbers of animals for beef, casein, chicken, fish, pork and soy protein groups are 11, 10, 11, 11, 11 and 10 respectively. The left histogram shows the LDA scores computed for features at the OTU level. The right heatmap shows the relative abundance of OTU (log 10 transformed). Each column represents one animal and each row represents the OTU corresponding to left one.

A pairwise comparison between the non-meat and the red meat protein classes indicated that 36 OTUs were significantly different (Fig. 4a and Supplementary Table S2 online). Twenty-two of these OTUs were higher in the non-meat protein class and fourteen OTUs were higher in red meat protein class (p < 0.01). The relative abundance of OTU434 (*Alloprevotella*) was higher in the non-meat protein class than in the red meat protein class (2.6% versus 0.22%, p < 0.01). OTU227 (*Roseburia*) was one of the most predominant bacteria in the non-meat protein class, but its abundance was much lower in the red meat protein class (7.1% versus 1.3%, p < 0.01). OTU66 (*Prevotellaceae uncultured*) was detected in the non-meat protein class (p < 0.001; 0.15% for the casein group and 1.19% for the soy group) but not in the red meat protein class.

A comparison between the non-meat and the white meat protein classes showed that 56 OTUs were significantly different (Fig. 4b and Supplementary Table S3 online). Thirty-three of those OTUs were higher in the non-meat protein class, but the other twenty-three OTUs were higher in white meat protein class. Again, OTU227 (*Roseburia*) and OTU66 (*Prevotellaceae uncultured*) were typical bacteria for the non-meat protein class. OTU560 (*Bacteroides*) can be considered a characteristic bacterium for non-meat protein class (0.21% versus 2.68%, p < 0.001). Five OTUs (OTU60, OTU149, OTU817, OTU522 and OTU437) that represent genus *Lactobacillus*, were more abundant in the white meat protein class.

In addition, one-hundred and five OTUs were significantly different between the red and white meat protein class (P < 0.05, Supplementary Fig. S3 and Table S4 online). Eighty-three OTUs were higher in the red meat protein class, and the other 22 were higher in the white meat protein class. There were only 16 OTUs that had significant diversity (p < 0.001, Fig. 4c). This indicated that gut bacteria may not show too many different responses to red and white meat proteins. The relative abundances of genus *Lactobacillus* (OTU60, OTU149, OTU817, and OTU437) were higher in the white meat protein class, but *Oscillibacter* (OTU276, OTU134, OTU491, and OTU726) were higher in the red meat protein class. A significant difference was also found in *Bacteroides* (OTU560) between the red and white meat protein classes (1% versus 0.21%, p < 0.001).

**Figure 5:**
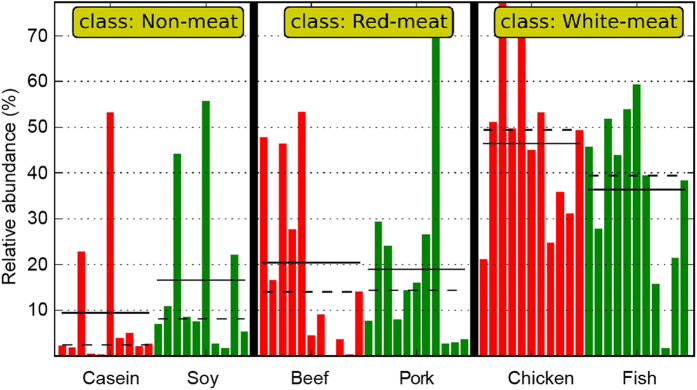
Relative abundance of *Lactobacillus* in rat caecum. The means and medians are shown as solid and dashed lines in each group. Each column represents one animal and there are totally 64 animals. The numbers of animals for beef, casein, chicken, fish, pork and soy protein groups are 11, 10, 11, 11, 11 and 10 respectively. These samples were further classified into non-meat protein class, red meat protein class and white meat protein class.

As shown above, genus *Lactobacillus* is a characteristic bacterium. A multiple comparison indicated that rats fed with chicken meat protein had the highest abundance of *Lactobacillus*, but it was the lowest for the casein group. In addition, rats fed with soy protein also showed a lower abundance of *Lactobacillus* than those fed with meat proteins (Fig. 5 and Table 1).

### Metastats analysis of gut bacteria

**Figure 6:**
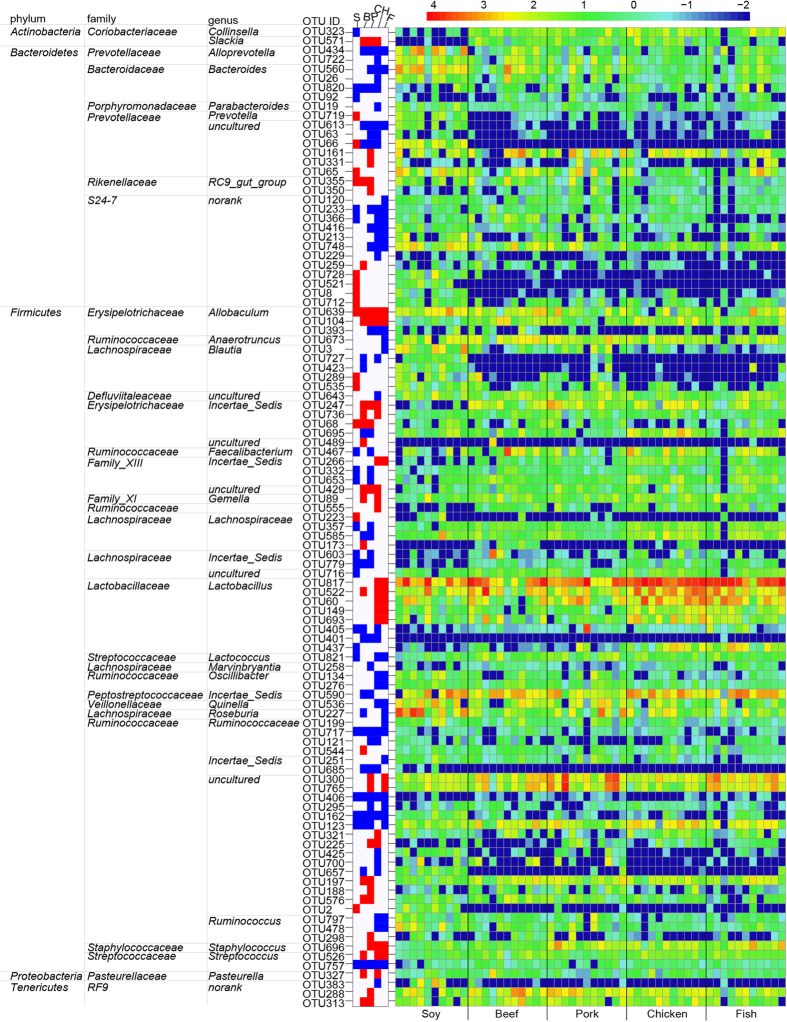
Heatmaps of gut bacteria at the OTU level using LefSe. The figure includes three parts. The right part shows the relative abundance (log 10 transformation) of OTU. Each column represents one animal and each row represents one OTU. The numbers of animals for beef, casein, chicken, fish, pork and soy protein groups are 11, 10, 11, 11, 11 and 10 respectively. The middle part shows the change folds of OTU that changed significantly (p < 0.05) when compared to casein group. Red denotes an increase while blue denotes a decrease. S, soy protein group; B, beef protein group; P, pork protein group; CH, chicken protein group; F, fish protein group. The left table lists significant difference of OTU and corresponding phyla, families and genera.

To identify the effect of dietary proteins on gut bacteria, we did a pairwise comparison using Metastats analysis on the casein group (as control, casein is the sole protein in standard rat diets recommended by the American Institute of Nutrition) and one of the other five groups. Metastats analysis was performed on those OTUs with a relative abundance that was at least more than 0.1% in one group. The results showed that the overall profile of gut bacteria in rat caecum differed significantly (P < 0.05) (Fig. 6 and Supplementary Table S5–S9 online). Meanwhile, individual animals showed a great variation in response to different dietary proteins. One hundred and five phylotypes were significantly different (p < 0.05) at least in one pairwise comparison. Seventy-one of these phylotypes belonged to the phylum *Firmicutes* and twenty-eight phylotypes to the phylum *Bacteroidetes*.

**Figure 7:**
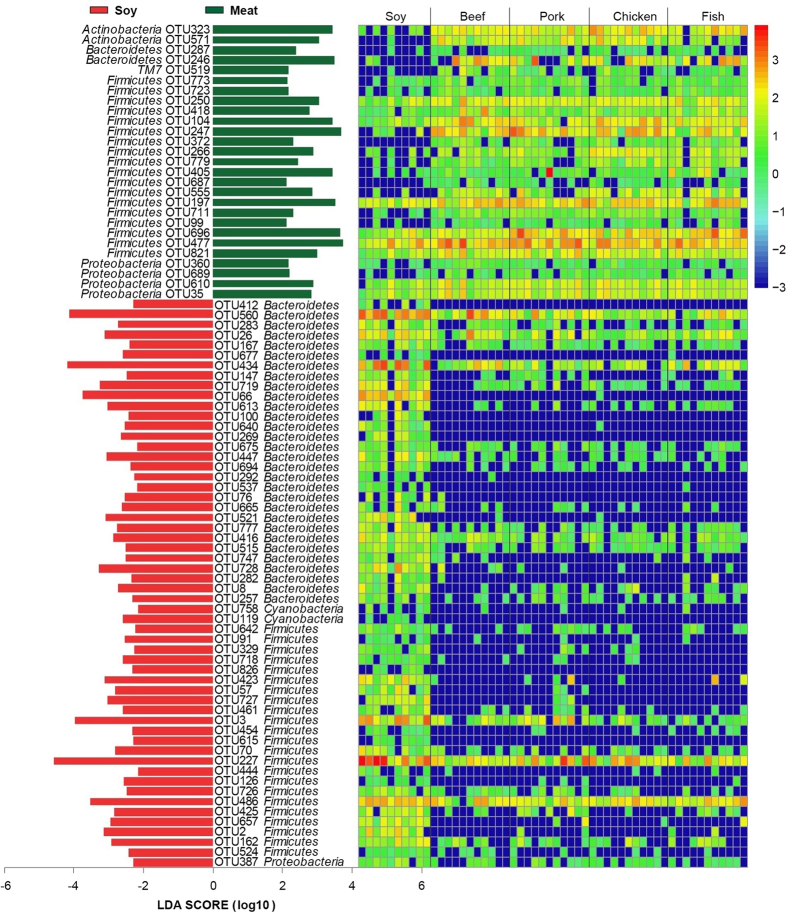
Comparisons of gut bacteria between meat protein class and soy protein group using LefSe. The numbers of animals for beef, chicken, fish, pork and soy protein groups are 11, 11, 11, 11, and 10 respectively. The left histogram shows the LDA scores computed for features at the OTU level. The right heatmap shows the relative abundance of OTU (log 10 transformed). Each column represents one animal and each row represents one OTU.

From the heatmap in Fig. 6, we found that rats fed with meat proteins had significantly different profiles of gut bacteria from those fed with soy protein. We merged all the meat protein groups as meat class, and did a LeFSe analysis between the meat and soy protein classes (Fig. 7 and Supplementary Table S10 online). The results showed that there were eighty-three OTUs for separating the meat class from soy protein group. The soy protein group had a greater relative abundance of *Bacteroidetes* than meat class.

### Antigen load from gut bacteria to the host

**Figure 8:**
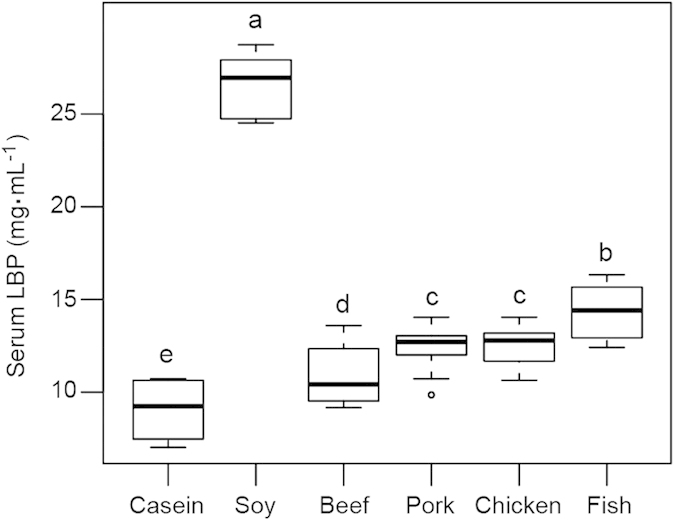
The serum levels of LBP in response to dietary proteins. Note: The numbers of animals for beef, casein, chicken, fish, pork and soy protein groups are 11, 10, 11, 11, 11, and 10 respectively. In a typical box plot, the top of the rectangle indicates the third quartile, a horizontal line near the middle of the rectangle indicates the median, and the bottom of the rectangle indicates the first quartile. A vertical line extends from the top of the rectangle to indicate the maximum value, and another vertical line extends from the bottom of the rectangle to indicate the minimum value. The relative vertical spacing between the labels reflects the values of the variable in proportion. The dot in pork protein group shows an outlier. The data were analyzed by one-way analysis of variance and means were compared by the procedure of Duncan’s multiple comparison. ^a,b,c,d^ Means with different superscripts differed significantly (p < 0.05).

The level of lipopolysaccharide-binding protein (LBP) in the host rat serum was analyzed to determine the antigen load derived from gut bacteria. Rats fed with soy protein had a higher LBP level (p < 0.001, Fig. 8) than those fed with other proteins, while the casein group had the lowest LBP level (P < 0.05). For the meat groups, the rats fed with fish protein had a higher LBP level (P < 0.05), whereas the red meat protein class was lower compared to white meat protein class.

### Growth performance

There was no significant difference in body weight between any two groups on day 0 (p > 0.05, Table 2). After 90-day feeding, the chicken protein group had a lower body weight and body weight gain, but the highest ratios of perirenal fat weight/body weight (P/W, %) and epididymis fat weight/body weight (E/W, %). Casein group had the highest body weight gain and fish protein group had the highest body weight and the highest ratio of P/W. Beef protein group had lower body weight gain and visceral content (E/W+P/W).

## Discussion

The mammalian gut bacteria can be considered as an efficient movable bioreactor. Gut bacteria have a close connection with the host through metabolite input to maintain energy homeostasis in the intestinal mucosa^19^. Although the composition of gut bacteria is relatively stable, it can be affected by many factors, especially the diet.

In the present study, our data showed different compositions of gut bacteria in rat caecum after feeding rats with different proteins. Compared with non-meat protein class, meat protein class, and in particular to white meat protein class, had a higher abundance of *Lactobacillus. Lactobacillus* has been considered as a key player in host metabolic balance^20^,^21^. A greater abundance of *Lactobacillus* may reduce the antigen load from gut bacteria to the host, and may alleviate certain inflammation responses and metabolic syndromes^22^,^23^,^24^,^25^. In other words, the intake of meat proteins at recommended level may be beneficial for the proliferation of commensal bacteria.

Lipopolysaccharide (LPS) is an endotoxin that is produced by Gram-negative bacteria^26^,^27^. If LPS enters the circulation system, it would upregulate the expression and translation of the binding protein (LBP) in liver^28^. LBP delivers LPS to CD14 and TLR4, and triggers the expression of pro-inflammatory cytokines, including TNF (tumor necrosis factor), interleukins 1 and 6 (IL-1 and IL-6)^29^. The LBP level in blood is usually considered as a biomarker for an inflammatory response and an antigen load to the host^30^. Our data showed that the intake of casein and meat proteins reduced serum LBP level when compared to soy protein. This may be associated with composition of the gut bacteria. *Bacteroidetes* has been shown to be the major lipopolysaccharide-producing bacterium in the gut^31^. The soy protein group had higher abundance of *Bacteroidetes* than the meat protein groups. Therefore, the intake of casein and meat proteins may maintain a more balanced composition of gut bacteria and reduce the antigen load and inflammatory response to the host.

The ratio of phyla *Firmicutes* to *Bacteroidetes* (the F/B ratio) in gut bacteria may have a certain relationship with obesity and other metabolic disorders^32^,^33^,^34^,^35^. Although F/B ratios varied greatly with diet, none of the rats showed any characteristics of metabolic disorders. Meat protein consumption has been shown to be positively associated with weight gain^36^, but a negative association was reported between the intake of plant protein and obesity^2^,^37^. In the present study, the soy protein group had a lower body weight but a higher visceral fat content than pork protein group. The beef protein group had a lower visceral content than the soy protein group. Thus, the intake of soy protein may affect body weight gain, but not fat deposition. This means that red meat proteins may decrease visceral fat and increase weight of other tissues as compared with soy protein. In fact, the ingestion of meat proteins can enhance energy expenditure, satiety and fat loss more than plant proteins^38^. However, our data showed a higher level of visceral fat for the chicken and fish protein groups when compared to the soy protein group. This difference may be explained by two aspects: Sprague-Dawley rats are less sensitive to dietary proteins than mice^39^, and/or the diverse structure of gut bacteria is a response to dietary proteins as a result of different growth performance.

As mentioned above, excessive intake of red meat may be a high risk of mortality for CRC^4^,^6^,^40^, which is characteristic of higher abundance of *Fusobacterium* and *Bacteroides*, and lower abundance of *Lactobacillus* and *Roseburia*^41^. The changes were found to be accompanied by a significant reduction in *Firmicutes* and *Bacteroidete*^42^. However, in the present study, we found higher *Lactobacillus* but lower *Bacteroides* in the meat protein groups. OTU227 (genus *Roseburia*) was numerically higher in the non-meat than in meat protein class, but the relative abundance of *Roseburia* was not significantly different among these groups. The meat protein groups had a higher abundance of *Firmicutes* but lower abundance of *Bacteroidetes* than the non-meat protein groups. These results did not indicate any relationship between the intake of meat proteins and CRC in terms of gut bacteria. The intake, cooking method and other components of red meat, such as heme iron, may be also important and should be considered when evaluating the relationship between meat or meat protein consumption and health concerns.

In conclusion, dietary proteins have a substantial influence on the composition of gut bacteria in the caecum. The specific phylotypes responding to dietary proteins might play a critical role in the health maintenance of the host. Our results suggest that the intake of dairy and meat proteins at recommended level may be beneficial to maintain a balanced composition of gut bacteria compared with soy protein. These findings provide a novel insight into the relationship between meat intake and host health in terms of gut bacteria, and indicate that intake of red meat or white meat proteins at recommended level may be more beneficial for health than non-meat proteins.

## Materials and methods

### Animals and diets

The experimental protocol was approved by the Ethical Committee of Experimental Animal Center of Nanjing Agricultural University in accordance with the National Guidelines for Experimental Animal Welfare (MOST of People’s Republic of China, 2006). A total of 66 male Sprague-Dawley rats (117 g ± 10 g) were purchased from Zhejiang Experimental Animal Center (Zhejiang, P.R.China, SCXK9 < Zhejiang > 2008-00) and housed in a specific pathogen-free animal center (SYXK < Jiangsu > 2011-0037). After 7-day acclimatization, the rats were assigned randomly to six formulated diets with proteins from pork, beef, chicken, fish, soy or casein. The animals were housed individually in plastic cages and given water and diets *ad libitum* in a temperature (20.0 ± 0.5 °C) - and humidity (60 ± 10%)-controlled room with a 12 h light-dark cycle.

Meat proteins were extracted from beef *longissimus dorsi* muscle, pork *longissimus dorsi* muscle, chicken *pectoralis major* muscle and fish muscle obtained from a local meat company (Sushi, Jiangsu). Visible fat and connective tissue were removed from beef, pork and chicken muscles, and scales and bones were removed from fish before protein extraction. The muscles were finely chopped and formed into a 2-cm cubes. The cubes were placed in plastic bags and cooked in a 72 °C water bath until the center temperature was 70 °C. During cooking, the center temperature was monitored using a digital thermometer fitted with a thin temperature probe (RM-113, Ruiming, Changzhou, China). The cooked meat was chilled, freeze-dried, and ground into powder. Intramuscular fat was removed by using a mixture of solvent methylene chloride/methanol (V/V = 2:1), and organic solvent was removed in a fume hood. Casein and soy proteins were obtained from Jiangsu Teluofei, Inc. (Nantong, China) and Linyi Shansong Biological Products Inc. (Linyi, China), respectively.

Animal diets were prepared according to the recommendation of the American Institute of Nutrition (AIN-93) to meet the nutritional requirements for growing rats^43^. The diets containing protein (20%), cornstarch (39.75%), dextrinized cornstarch (13.2%), sucrose (10%), soybean oil (7%), fiber (5%), mineral mix (3.5%), vitamin mix (1%), L-cystine (0.3%), choline bitartrate (0.25%) and tert-butylhydroquinone (0.0014%), were prepared by Jiangsu Teluofei, Inc. (Nantong, China).

### Caecal sample collection and DNA extraction

After 90-day feeding, all rats were decapitalized after 4 h fasting. The caecal contents were collected, frozen in liquid nitrogen, and stored at −80 °C before being analyzed. DNA was extracted from each sample using the QIAamp DNA Stool Mini Kit (NO.51504, Qiagen, Germany) according to the manufacturer’s protocol.

### Amplification and high throughput sequencing of gut bacteria

The 16S ribosomal RNA (rRNA) gene from caecal contents was amplified with universal primers: F 515 (5′-GTGCCAGCMGCCGCGG-3′) and R 907 (5′-CCGTCAATTCMTTTRAGTTT-3′). The V4-V5 hypervariable region that is universal for nearly all bacterial taxa^44^ was applied for amplification. PCR reactions were run in a 20 μL thermocycler PCR system (GeneAmp® 9700, ABI, USA) with following program 2min of denaturation at 95 °C, 25 cycles of 30 s at 95 °C (denaturation), 30 s for annealing (1 °C reduced for every two cycles from 65 to 57 °C, followed by one cycle at 56 °C and one cycle at 55 °C), 1 min at 72 °C (elongation), and a final extension at 72 °C for 10 min. The reaction mixture contained 4 μL of 5 × FastPfu Buffer, 2 μL of 2.5 mM dNTPs, 0.8 μL of each primer (5 μM), 0.4 μL of FastPfu Polymerase (TransGen Biotech, Beijing, China), and 10 ng of template DNA.

Amplicons were extracted from 2% agarose gels and purified using the AxyPrep DNA Gel Extraction Kit (Axygen Biosciences, Union City, CA, U.S.) according to the manufacturer’s protocol and quantified using QuantiFluor™ -ST (Promega, U.S.). Purified amplicons were sequenced under the MiSeq platform (Illumina, San Diego, California, USA) according to the standard protocols in a commercial company (Shanghai Majorbio Bio-Pharm Technology Co., Ltd, Shanghai, China).

### Serum LBP

Blood samples were collected from the eyeballs of rats after 4 h fasting and centrifuged at 12,000 × g for 30 min to pellet the blood cells. Serum samples were collected and stored at −80 °C until required for analyses. The serum LBP levels of samples were determined using a commercial ELISA Kit (No. MBS703266, MyBioSource, Inc. San Diego, California, USA) according to the manufacturer’s protocol.

### Bioinformatics and statistical analyses

Raw fastq files were de-multiplexed and quality filtered using QIIME (version 1.17) with the following criteria: 1) the 250 bp reads were truncated at any site receiving an average quality score <20 over a 10 bp sliding window; 2) the truncated reads less than 50 bp were removed; 3) the specific barcodes were exactly matched; 4) the mismatching part with primers allowed was less than 2 bp; 5) reads containing ambiguous characters were removed; 6) only sequences that overlapped by more than 10 bp were assembled according to their overlap sequence; 7) reads that could not be assembled were discarded. Operational Taxonomic Units (OTU) were clustered with 97% similarity cutoff using UPARSE (version 7.1 http://drive5.com/uparse/) and chimeric sequences were identified and removed using UCHIME. RDP classifier^45^ was used for taxonomical assignments of each sequence at 70% confidence level using 16S rRNA sequences from Silva release 119 (http://www.arb-silva.de)^46^. Rarefaction analysis^47^ and alpha diversities^48^ were performed using Mothur (version v.1.30.1, http://www.mothur.org). Community richness was evaluated by Chao and ACE. Community diversity was evaluated by Shannon index and Simpson index. The Good’s coverage analysis was evaluated. Bray Curtis similarity clustering analysis was performed by R package (R 3.0.2, http://cran.r-project.org/).

LEfSe analysis was performed (http://huttenhower.sph.harvard.edu/galaxy/) to find the highly-dimensional gut bacteria and characterize the differences between two or more biological conditions (or classes)^49^. The differences in features were identified at the OTU level. The six groups were categorized into three classes: red meat (beef and pork), white meat (chicken and fish) and non-meat (soybean and casein). The LEfSe analysis conditions were as follows: 1) alpha value for the factorial Kruskal-Wallis test among classes was less than 0.05; 2) alpha value for the pairwise Wilcoxon test among subclasses was less than 0.05; 3) the threshold on the logarithmic LDA score for discriminative features was less than 2.0; 4) multi-class analysis was set as all-against-all.

Differentially abundant features of bacterial taxa at the OTU level were performed using Metastats (http://metastats.cbcb.umd.edu/), which is a statistically strict method designed specifically to compare microbial communities on 16S rRNA abundance data^50^.

Differences in serum LBP level and relative abundance of bacteria among six groups were evaluated by one-way analysis of variance and Bartlett’s test, and means were compared by Duncan’s multiple comparison using SAS system (version 9.2), and p value less than 0.05 was declared significant.

## Additional Information

**How to cite this article**: Zhu, Y. *et al.* Meat, dairy and plant proteins alter bacterial composition of rat gut bacteria. *Sci. Rep.* **5**, 15220; doi: 10.1038/srep15220 (2015).

## Supplementary Material

Supplementary Information

## Figures and Tables

**Table 1:** Relative abundance of *Lactobcillus* in rat caecum.

Groups	Relative abundance (%)	Median (%)	Range (%)
Chicken (n^1^ = 11)	46.41^a^	49.39	21.20–77.28
Fish (n = 11)	36.3^ab^	39.44	1.73–59.31
Beef (n = 11)	20.32^bc^	13.99	0.13–53.30
Pork (n = 11)	18.88^c^	14.38	2.72–72.37
Soybean (n = 10)	16.6^c^	7.6	1.75–55.65
Casein (n = 10)	9.45^c^	2.24	0.31–53.22

Note: The data were analyzed by one-way analysis of variance and means were compared by Duncan’s multiple comparison.

^1^‘n’ is the number of animals in each group. ^a,b,c^Means with different superscripts differed significantly (p < 0.05).

**Table 2:** Growth performance of rats in response to dietary proteins.

	Casein (n^1^ = 10)	Soybean (n = 10)	Fish (n = 11)	Chicken (n = 11)	Pork (n = 11)	Beef (n = 11)
BW (0d)	164.91 ± 15.45^a^	167.6 ± 12.32^a^	171.45 ± 10.32^a^	167.1 ± 11.18^a^	167.82 ± 14.74^a^	168.55 ± 15.15^a^
BW (90d)	666.44 ± 79.94^ab^	630.20 ± 61.69^bc^	686.44 ± 43.25^a^	650.91 ± 76.47^abc^	643.82 ± 41.28^bc^	610.00 ± 70.05^c^
BWG	523.40 ± 91.99^a^	467.40 ± 60.80^abc^	514.44 ± 45.83^ab^	457.89 ± 34.83^bc^	473.33 ± 25.71^abc^	435.14 ± 64.48^c^
P/W (%)	4.61 ± 0.89^a^	4.20 ± 0.84^ab^	5.10 ± 1.41^a^	4.88 ± 1.04^a^	4.13 ± 0.71^ab^	3.38 ± 0.98^b^
E/W (%)	3.38 ± 0.69^ab^	2.58 ± 0.51^cd^	2.95 ± 0.31^bc^	3.52 ± 0.31^a^	2.11 ± 0.25^e^	2.48 ± 0.53^de^

Note: The data were analyzed by one-way analysis of variance and means were compared by Duncan’s multiple comparison.

^1^‘n’ is the number of animalsc in each group. ^a,b,c,d,e^Means with different superscripts differed significantly (p < 0.05). BW: body weight; BWG: body weight gain; P/W: perirenal fat weight/body weight; E/W: epididymis fat weight/body weight.
