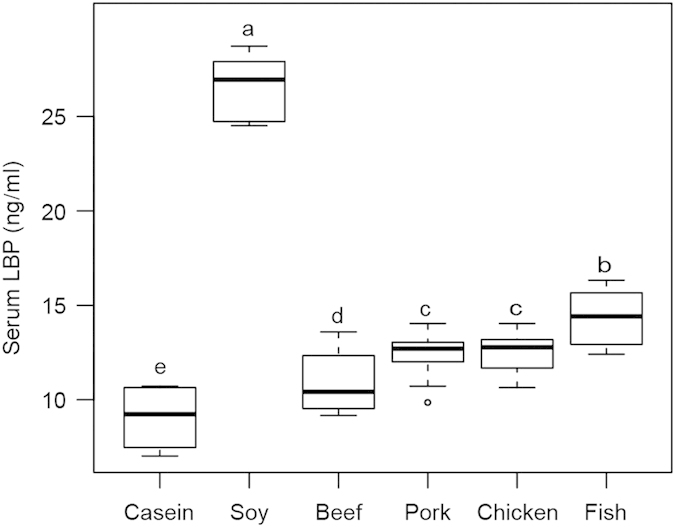# Erratum: Meat, dairy and plant proteins alter bacterial composition of rat gut bacteria

**DOI:** 10.1038/srep16546

**Published:** 2015-11-17

**Authors:** Yingying Zhu, Xisha Lin, Fan Zhao, Xuebin Shi, He Li, Yingqiu Li, Weiyun Zhu, Xinglian Xu, Chunbao Li, Guanghong Zhou

Scientific Reports 5: Article number: 15220; 10.1038/srep15220published online: 10142015; updated: 11172015

The original version of this Article contained a typographical error in the spelling of the author Chunbao Li, which was incorrectly given as Chunbao Lu.

In addition, there was an error in Figure 8, where the y-axis label ‘Serum LBP (ng/ml)’ was incorrectly given as ‘Serum LBP (mg×ml-1). The correct Figure 8 appears below as Figure 1. These errors have now been corrected in the PDF and HTML versions of the Article.

## Figures and Tables

**Figure 1:**