# Supplementary material for: Meat, dairy and plant proteins alter bacterial composition of rat gut bacteria
Source: Sci Rep. 2015 Oct 14;5:15220. doi: 10.1038/srep15220 (PMC4604471; doi:10.1038/srep15220)
Supplement: Supplementary Information [file srep15220-s1.pdf]

Meat, dairy and plant proteins alter bacterial composition of rat gut bacteria

Yingying Zhu[a,1], Chunbao Li[a,1], Xisha Lin[a], Fan Zhao[a], Xuebin Shi[a], He Li[a], Yingqiu Li[a], Weiyun Zhu[b], Xinglian Xu[a], Guanghong Zhou[a,]*

[a] Key Laboratory of Meat Processing and Quality Control, MOE; Key Laboratory of Animal Products Processing, MOA; Jiang Synergetic Innovation Center of Meat Processing and Quality Control; Synergetic Innovation Center of Food Safety and Nutrition; Nanjing Agricultural University; Nanjing 210095, P.R. China

[b] Gastrointestinal Microbiology Joint Research Center; Laboratory of Gastrointestinal Microbiology; Nanjing Agricultural University; Nanjing 210095, P.R. China

[1] Equally contributed.

*Corresponding author

Dr. Guanghong Zhou

E-mail: guanghong.zhou@hotmail.com;

Tel: 86 25 84395376; Fax: 86 25 84395679

Running title: Gut bacteria in response to protein source

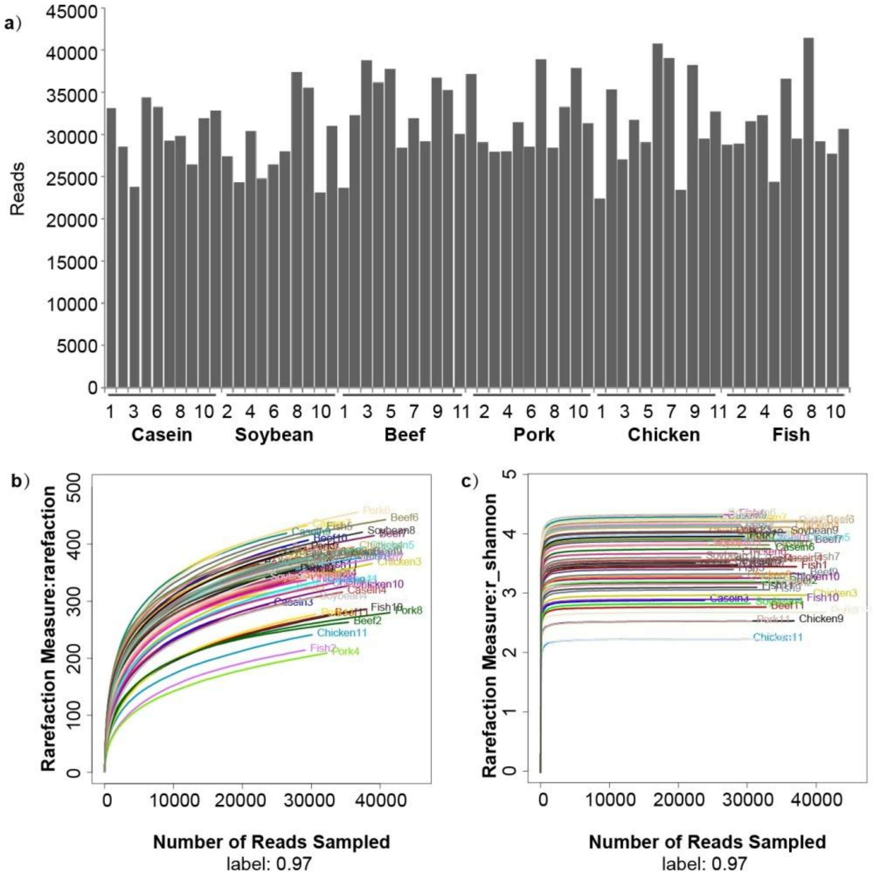

Supplementary Figure S1 Diversity estimation of caecal microbiota in all samples.

a) The number of usable raw reads. Each bar represents one animal;

b) Rarefaction curves. Each line represents one animal;

d) Shannon–Wiener diversity index curves. Each line represents one animal.

Note: there are totally 64 animals, of which 10 from casein protein group, 10 from soy protein group, 11 from beef protein group, 11 from pork protein group, 11 from chicken protein group and 11 from fish protein group.

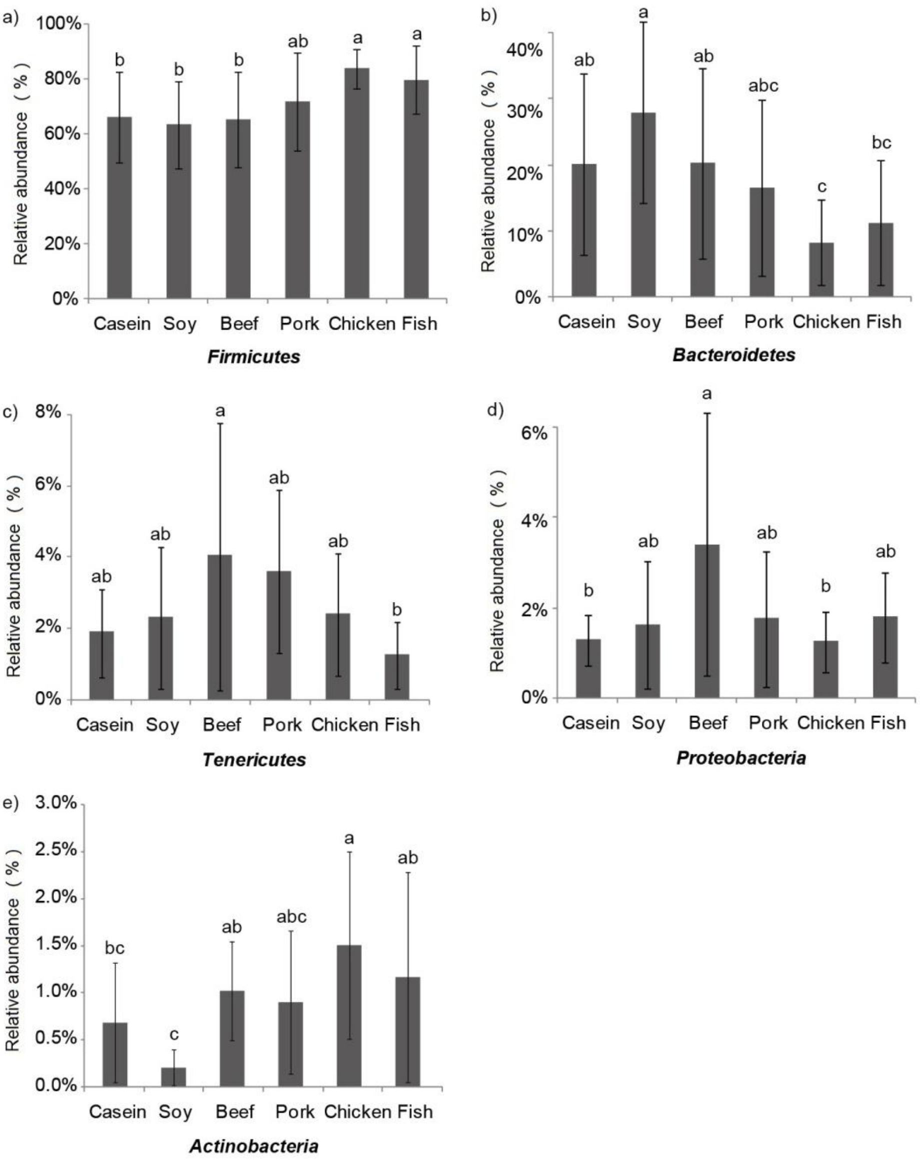

Supplementary Figure S2 Changes of relative abundance of several significantly different phyla during the trial.

a) *Firmicutes*; b) *Bacteroidetes*, c) *Tenericutes*; d) *Proteobacteria*; and e) *Actinobacteria*. Data are shown as means ± standard deviation. The data were analyzed by one-way analysis of variance and means were compared by the procedure of Duncan's multiple. [a,b,c] Means with different superscripts differed significantly ($p<0.05$). There are totally 64 animals, of which 10 from casein protein group, 10 from soy protein group, 11 from beef protein group, 11 from pork protein group, 11 from chicken protein group and 11 from fish protein group.

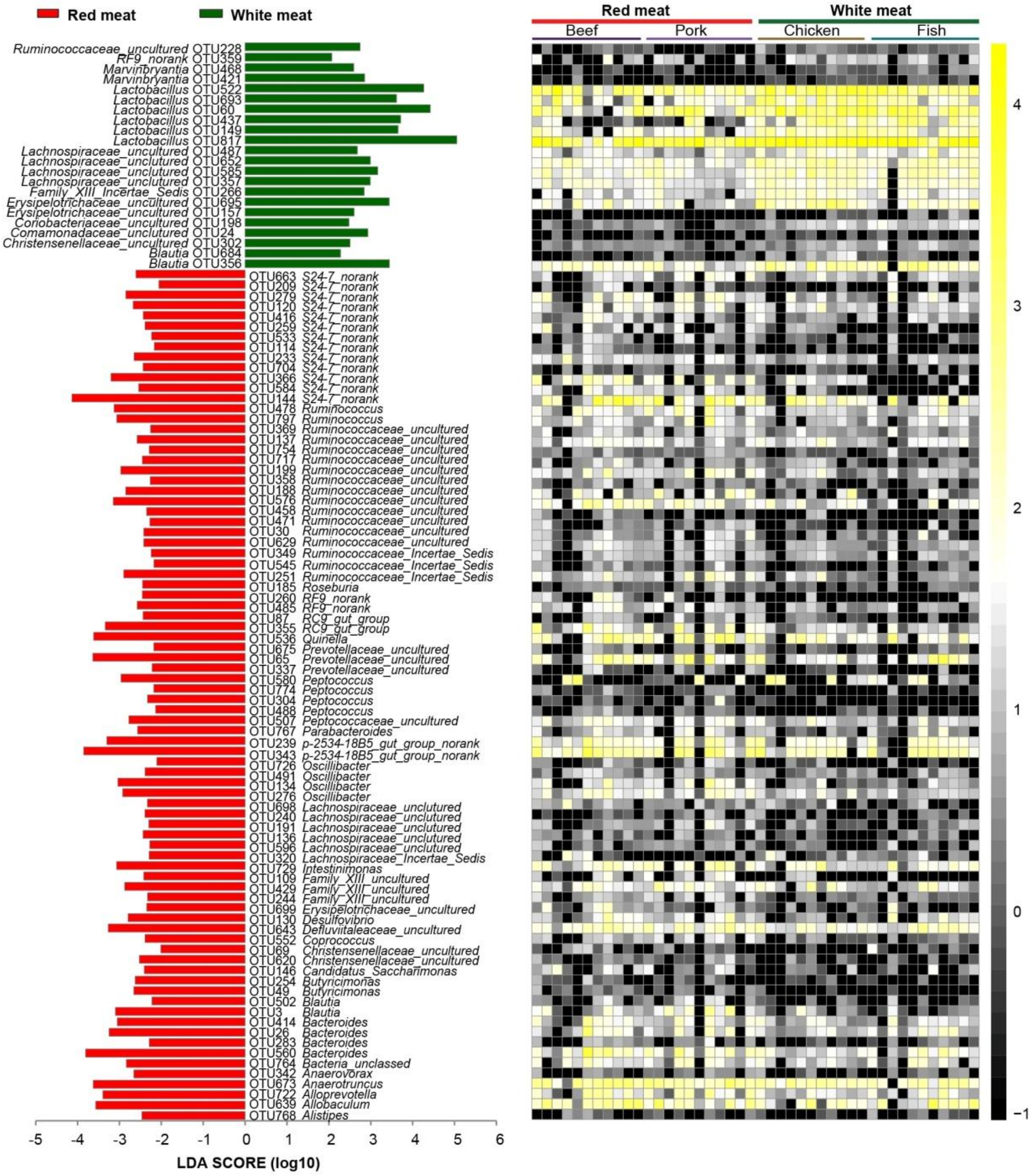

Supplementary Figure S3 Comparisons of caecal bacterial community in response to red meat class and white meat class using LefSe.

The left histogram shows the LDA scores computed for features at the OTU level that distinguished between two classes. The right heatmap shows the relative abundance of OTU (log 10 transformed). Each column represents a animal and each row represents one OTU corresponding to left one. The numbers of animals for beef, chicken, fish, and pork protein groups are 11, 11, 11 and 11 respectively.

Supplementary Table S1 Richness and diversity indexes relative to each sample.

Sample ID	Reads	0.97					
		OTU Number	ACE	Chao	Coverage	Shannon	Simpson
Soy10	23284	340	489 (450,541)	471 415,567	99.6%	3.62 (3.6,3.65)	0.0783 (0.0759,0.0807)
Soy11	31131	335	480 (441,533)	411 379,467	99.7%	3.23 (3.21,3.25)	0.1551 (0.1513,0.159)
Soy2	27455	342	390 (371,421)	392 369,436	99.8%	3.55 (3.53,3.57)	0.1003 (0.0974,0.1031)
Soy3	24292	368	446 (418,488)	457 419,523	99.6%	3.84 (3.82,3.87)	0.0533 (0.0519,0.0547)
Soy4	30466	306	371 (347,410)	399 357,474	99.8%	2.85 (2.83,2.87)	0.166 (0.1626,0.1695)
Soy5	24870	384	435 (415,468)	445 417,498	99.7%	4.26 (4.24,4.28)	0.0313 (0.0306,0.0321)
Soy6	26498	392	458 (434,496)	465 433,522	99.7%	4.35 (4.33,4.37)	0.025 (0.0245,0.0255)
Soy7	28055	344	411 (386,449)	410 381,462	99.7%	3.51 (3.49,3.53)	0.0756 (0.0739,0.0773)
Soy8	37429	421	504 (475,549)	524 481,599	99.8%	4.26 (4.24,4.28)	0.032 (0.0312,0.0327)
Soy9	35559	383	432 (413,464)	424 405,460	99.8%	4.05 (4.03,4.06)	0.0378 (0.0371,0.0384)
Casein1	33202	376	455 (427,499)	479 434,556	99.7%	3.84 (3.82,3.86)	0.0611 (0.0595,0.0628)
Casein10	31913	383	457 (430,500)	471 432,541	99.8%	4.03 (4.01,4.05)	0.0475 (0.0463,0.0487)
Casein11	32861	382	448 (423,488)	478 435,556	99.8%	3.94 (3.92,3.96)	0.0539 (0.0525,0.0552)
Casein2	28622	347	418 (392,458)	433 395,500	99.7%	3.5 (3.48,3.52)	0.0797 (0.0779,0.0815)
Casein3	23839	295	358 (334,396)	372 337,436	99.7%	2.9 (2.88,2.93)	0.196 (0.1909,0.2011)
Casein4	34520	316	382 (357,423)	398 361,466	99.8%	3.58 (3.57,3.6)	0.0567 (0.0557,0.0577)
Casein6	33379	384	457 (431,499)	469 432,535	99.8%	3.77 (3.75,3.79)	0.0666 (0.0651,0.0681)
Casein7	29395	433	497 (474,533)	489 464,531	99.7%	4.27 (4.25,4.29)	0.0358 (0.0348,0.0368)
Casein8	29838	347	423 (395,468)	436 396,508	99.7%	3.96 (3.94,3.98)	0.0399 (0.0392,0.0407)
Casein9	26423	419	486 (462,523)	482 455,530	99.7%	4.31 (4.29,4.33)	0.0297 (0.029,0.0304)

Chicken1	23698	367	419<br>(399,451)	422<br>397,467	99.7%	4.03<br>(4.01,4.05)	0.0419<br>(0.0408,0.0431)
Chicken10	35386	327	404<br>(376,447)	394<br>365,445	99.8%	3.28<br>(3.26,3.3)	0.1005<br>(0.0985,0.1025)
Chicken11	30127	241	375<br>(337,428)	329<br>289,403	99.8%	2.25<br>(2.23,2.28)	0.2863<br>(0.2815,0.291)
Chicken2	32294	329	402<br>(376,445)	445<br>393,537	99.7%	3.18<br>(3.16,3.2)	0.1204<br>(0.1178,0.123)
Chicken3	38834	366	461<br>(428,510)	478<br>432,557	99.8%	2.99<br>(2.97,3.01)	0.1727<br>(0.1693,0.1761)
Chicken4	36238	395	472<br>(445,515)	469<br>437,524	99.8%	4.13<br>(4.12,4.15)	0.0378<br>(0.0368,0.0387)
Chicken5	37778	395	459<br>(436,495)	467<br>436,522	99.8%	3.93<br>(3.91,3.94)	0.0432<br>(0.0424,0.044)
Chicken6	28520	342	407<br>(383,445)	398<br>373,442	99.7%	3.69<br>(3.67,3.71)	0.0744<br>(0.0723,0.0764)
Chicken7	31958	333	401<br>(376,440)	429<br>387,505	99.8%	3.47<br>(3.45,3.49)	0.0865<br>(0.0844,0.0886)
Chicken8	29262	337	394<br>(372,428)	390<br>366,434	99.8%	3.31<br>(3.29,3.33)	0.1101<br>(0.1077,0.1126)
Chicken9	36811	298	379<br>(350,426)	383<br>346,448	99.8%	2.56<br>(2.54,2.58)	0.1923<br>(0.1891,0.1955)
Fish1	37163	376	431<br>(410,464)	430<br>405,474	99.8%	3.47<br>(3.45,3.49)	0.0994<br>(0.0971,0.1017)
Fish10	37909	286	422<br>(385,475)	377<br>337,448	99.8%	2.92<br>(2.9,2.94)	0.1239<br>(0.1218,0.1261)
Fish11	31402	361	450<br>(419,497)	499<br>441,600	99.7%	3.12<br>(3.1,3.15)	0.1562<br>(0.1527,0.1597)
Fish2	29097	214	280<br>(254,325)	284<br>251,347	99.8%	2.47<br>(2.45,2.49)	0.1758<br>(0.1725,0.1791)
Fish3	27993	345	414<br>(388,453)	426<br>391,490	99.7%	3.42<br>(3.4,3.45)	0.1031<br>(0.1005,0.1057)
Fish4	28068	377	438<br>(415,475)	459<br>422,527	99.7%	4.35<br>(4.33,4.36)	0.0276<br>(0.0269,0.0284)
Fish5	31481	428	506<br>(479,548)	524<br>483,593	99.7%	4.38<br>(4.36,4.4)	0.0256<br>(0.0251,0.0262)
Fish6	28558	354	400<br>(382,430)	402<br>380,446	99.8%	4.02<br>(4,4.04)	0.0483<br>(0.0469,0.0497)
Fish7	38961	374	440<br>(416,478)	433<br>407,480	99.8%	3.61<br>(3.59,3.62)	0.0922<br>(0.09,0.0945)
Fish8	28424	378	454<br>(427,495)	449<br>419,501	99.7%	3.61<br>(3.59,3.63)	0.0805<br>(0.0784,0.0825)
Fish9	33283	332	396<br>(372,433)	416<br>379,484	99.8%	3.08<br>(3.06,3.1)	0.1663<br>(0.1625,0.1701)

Pork1	28829	365	430 (406,469)	438 405,497	99.7%	4.06 (4.05,4.08)	0.0374 (0.0366,0.0383)
Pork10	27669	355	433 (405,477)	458 413,537	99.7%	4.06 (4.04,4.08)	0.0325 (0.0319,0.0331)
Pork11	30675	277	372 (337,426)	376 332,453	99.7%	2.55 (2.53,2.57)	0.1838 (0.1808,0.1867)
Pork2	28973	353	427 (400,471)	439 401,507	99.7%	4.16 (4.14,4.17)	0.0317 (0.031,0.0324)
Pork3	31594	383	457 (430,499)	464 429,526	99.7%	4 (3.98,4.02)	0.0449 (0.0439,0.0459)
Pork4	32287	209	327 (292,377)	301 258,383	99.8%	2.25 (2.23,2.27)	0.2236 (0.22,0.2271)
Pork5	24517	378	455 (428,497)	492 443,579	99.6%	3.84 (3.81,3.86)	0.0602 (0.0585,0.0619)
Pork6	36770	456	535 (508,575)	538 504,595	99.8%	4.22 (4.2,4.24)	0.0371 (0.0362,0.038)
Pork7	29595	378	447 (422,486)	466 427,535	99.7%	3.98 (3.96,4)	0.0522 (0.0507,0.0536)
Pork8	41439	280	334 (312,371)	361 322,436	99.8%	2.7 (2.68,2.72)	0.1438 (0.142,0.1456)
Pork9	29210	394	483 (452,530)	476 442,533	99.7%	3.93 (3.91,3.95)	0.0553 (0.0538,0.0569)
Beef1	22450	365	423 (401,457)	431 401,484	99.7%	3.53 (3.5,3.55)	0.1309 (0.1268,0.1349)
Beef10	29585	407	486 (459,529)	492 456,553	99.7%	4.01 (3.99,4.03)	0.0656 (0.0635,0.0677)
Beef11	32736	277	361 (329,412)	385 337,474	99.8%	2.79 (2.77,2.81)	0.1409 (0.1383,0.1435)
Beef2	35417	263	302 (285,332)	323 293,385	99.9%	3.21 (3.19,3.22)	0.0908 (0.0891,0.0925)
Beef3	27105	322	364 (347,395)	389 356,453	99.8%	3.98 (3.97,4)	0.0378 (0.037,0.0386)
Beef4	31844	347	406 (384,442)	406 380,455	99.8%	3.58 (3.56,3.6)	0.0776 (0.0757,0.0794)
Beef5	29058	370	441 (415,482)	443 411,500	99.7%	4.18 (4.16,4.2)	0.0329 (0.0321,0.0337)
Beef6	40775	443	513 (488,551)	526 491,587	99.8%	4.23 (4.22,4.25)	0.0334 (0.0327,0.034)
Beef7	39137	415	497 (468,541)	513 472,586	99.8%	3.9 (3.89,3.92)	0.0503 (0.0493,0.0513)
Beef8	23512	349	418 (393,458)	456 409,539	99.7%	3.92 (3.9,3.94)	0.042 (0.041,0.043)
Beef9	38378	385	458 (432,498)	441 417,484	99.8%	3.35 (3.33,3.37)	0.1385 (0.1353,0.1417)

Supplementary Table S2 The differentially caecal bacterial communities between non meat and red meat class using LEfSe at the OTU level.

OTU ID	Toxon			Riched class	LDA Score (log10)	p value
	Phylum	Family	Genus			
OTU447	*Bacteroidetes*	*Rikenellaceae*	*Alistipes*	non meat	2.78	2.10E-04
OTU434	*Bacteroidetes*	*Prevotellaceae*	*Alloprevotella*	non meat	4.06	0.002
OTU635	*Proteobacteria*	*Desulfovibrionaceae*	*Desulfovibrio*	non meat	2.87	0.005
OTU193	*Proteobacteria*	*Helicobacteraceae*	*Helicobacter*	non meat	2.69	0.002
OTU786	*Firmicutes*	*Erysipelotrichaceae*	*Incertae_Sedis*	non meat	2.89	0.002
OTU454	*Firmicutes*	*Lachnospiraceae*	*Oribacterium*	non meat	3.00	2.07E-06
OTU479	*Bacteroidetes*	*Porphyromonadaceae*	*Parabacteroides*	non meat	2.62	9.69E-04
OTU147	*Bacteroidetes*	*Prevotellaceae*	*Prevotella*	non meat	2.56	4.03E-05
OTU719	*Bacteroidetes*	*Prevotellaceae*	*Prevotella*	non meat	2.93	5.37E-05
OTU66	*Bacteroidetes*	*Prevotellaceae*	*uncultured*	non meat	3.48	8.58E-09
OTU781	*Bacteroidetes*	*Prevotellaceae*	*uncultured*	non meat	2.58	0.002
OTU613	*Bacteroidetes*	*Prevotellaceae*	*uncultured*	non meat	3.10	1.80E-06
OTU70	*Firmicutes*	*Lachnospiraceae*	*Roseburia*	non meat	2.70	4.19E-04
OTU227	*Firmicutes*	*Lachnospiraceae*	*Roseburia*	non meat	4.45	0.001
OTU657	*Firmicutes*	*Ruminococcaceae*	*uncultured*	non meat	3.04	3.70E-06
OTU162	*Firmicutes*	*Ruminococcaceae*	*uncultured*	non meat	3.30	5.42E-07
OTU728	*Bacteroidetes*	*S24-7*	*norank*	non meat	2.99	1.66E-05
OTU282	*Bacteroidetes*	*S24-7*	*norank*	non meat	2.68	1.10E-05
OTU777	*Bacteroidetes*	*S24-7*	*norank*	non meat	2.74	4.09E-05
OTU515	*Bacteroidetes*	*S24-7*	*norank*	non meat	2.71	1.99E-04
OTU747	*Bacteroidetes*	*S24-7*	*norank*	non meat	2.74	3.21E-06
OTU387	*Proteobacteria*	*Alcaligenaceae*	*Sutterella*	non meat	2.69	0.005
OTU104	*Firmicutes*	*Erysipelotrichaceae*	*Allobaculum*	red meat	3.37	1.05E-04
OTU49	*Bacteroidetes*	*Porphyromonadaceae*	*Butyricimonas*	red meat	3.03	1.04E-05
OTU519	*TM7*			red meat	2.60	2.79E-04
OTU69	*Firmicutes*	*Christensenellaceae*	*uncultured*	red meat	2.99	5.26E-04
OTU250	*Firmicutes*	*Christensenellaceae*	*uncultured*	red meat	2.99	8.86E-04
OTU372	*Firmicutes*	*Eubacteriaceae*	*Eubacterium*	red meat	2.65	1.44E-06
OTU429	*Firmicutes*	*Family_XIII*	*uncultured*	red meat	2.98	9.14E-05
OTU247	*Firmicutes*	*Erysipelotrichaceae*	*Incertae_Sedis*	red meat	3.76	1.96E-06
OTU457	*Firmicutes*	*Family_XIII*	*Mogibacterium*	red meat	2.69	6.73E-04
OTU197	*Firmicutes*	*Ruminococcaceae*	*uncultured*	red meat	3.55	8.10E-04
OTU711	*Firmicutes*	*Ruminococcaceae*	*uncultured*	red meat	2.59	3.84E-05
OTU188	*Firmicutes*	*Ruminococcaceae*	*uncultured*	red meat	2.92	0.001
OTU571	*Actinobacteria*	*Coriobacteriaceae*	*Slackia*	red meat	3.10	7.07E-09
OTU696	*Firmicutes*	*Staphylococcaceae*	*Staphylococcus*	red meat	3.64	1.29E-04

Supplementary Table S3 The differentially caecal bacterial communities between non meat and white meat class using LEfSe at the OTU level.

OTU ID	Toxon			Riched class	LDA Score (log10)	p value
	Phylum	Family	Genus			
OTU447	*Bacteroidetes*	*Rikenellaceae*	*Alistipes*	non meat	2.77	9.18E-05
OTU434	*Bacteroidetes*	*Prevotellaceae*	*Alloprevotella*	non meat	4.08	0.001
OTU722	*Bacteroidetes*	*Prevotellaceae*	*Alloprevotella*	non meat	3.51	2.60E-04
OTU560	*Bacteroidetes*	*Bacteroidaceae*	*Bacteroides*	non meat	4.07	4.57E-06
OTU552	*Firmicutes*	*Lachnospiraceae*	*Coprococcus*	non meat	2.59	4.10E-04
OTU251	*Firmicutes*	*Ruminococcaceae*	*Incertae_Sedis*	non meat	2.93	1.28E-04
OTU454	*Firmicutes*	*Lachnospiraceae*	*Oribacterium*	non meat	2.91	7.95E-05
OTU134	*Firmicutes*	*Ruminococcaceae*	*Oscillibacter*	non meat	3.13	9.24E-06
OTU276	*Firmicutes*	*Ruminococcaceae*	*Oscillibacter*	non meat	2.98	1.58E-04
OTU726	*Firmicutes*	*Ruminococcaceae*	*Oscillibacter*	non meat	2.62	3.36E-04
OTU147	*Bacteroidetes*	*Prevotellaceae*	*Prevotella*	non meat	2.70	1.82E-06
OTU719	*Bacteroidetes*	*Prevotellaceae*	*Prevotella*	non meat	2.96	4.51E-06
OTU613	*Bacteroidetes*	*Prevotellaceae*	*uncultured*	non meat	3.08	1.48E-05
OTU66	*Bacteroidetes*	*Prevotellaceae*	*uncultured*	non meat	3.48	1.40E-08
OTU536	*Firmicutes*	*Veillonellaceae*	*Quinella*	non meat	3.81	0.003
OTU227	*Firmicutes*	*Lachnospiraceae*	*Roseburia*	non meat	4.52	0.002
OTU70	*Firmicutes*	*Lachnospiraceae*	*Roseburia*	non meat	2.72	9.10E-05
OTU199	*Firmicutes*	*Ruminococcaceae*	*Ruminococcaceae*	non meat	2.98	0.003
OTU27	*Firmicutes*	*Ruminococcaceae*	*Anaerotruncus*	non meat	2.67	6.55E-04
OTU162	*Firmicutes*	*Ruminococcaceae*	*uncultured*	non meat	3.29	3.08E-06
OTU657	*Firmicutes*	*Ruminococcaceae*	*uncultured*	non meat	3.04	2.48E-06
OTU478	*Firmicutes*	*Ruminococcaceae*	*Ruminococcus*	non meat	3.46	0.004
OTU797	*Firmicutes*	*Ruminococcaceae*	*Ruminococcus*	non meat	3.13	0.001
OTU114	*Bacteroidetes*	*S24-7*	*S24-7_norank*	non meat	2.71	1.66E-05
OTU213	*Bacteroidetes*	*S24-7*	*S24-7_norank*	non meat	3.50	0.004
OTU416	*Bacteroidetes*	*S24-7*	*S24-7_norank*	non meat	2.95	1.71E-04
OTU515	*Bacteroidetes*	*S24-7*	*S24-7_norank*	non meat	2.58	1.57E-04
OTU523	*Bacteroidetes*	*S24-7*	*S24-7_norank*	non meat	2.57	2.22E-04
OTU728	*Bacteroidetes*	*S24-7*	*S24-7_norank*	non meat	3.04	4.64E-07
OTU747	*Bacteroidetes*	*S24-7*	*S24-7_norank*	non meat	2.69	7.59E-06
OTU748	*Bacteroidetes*	*S24-7*	*S24-7_norank*	non meat	4.05	2.08E-05
OTU777	*Bacteroidetes*	*S24-7*	*S24-7_norank*	non meat	2.73	1.75E-05
OTU387	*Proteobacteria*	*Alcaligenaceae*	*Sutterella*	non meat	2.76	2.20E-04
OTU104	*Firmicutes*	*Erysipelotrichaceae*	*Allobaculum*	white meat	3.45	1.33E-06
OTU404	*Firmicutes*	*Lachnospiraceae*	*Blautia*	white meat	2.70	1.46E-05
OTU519	*TM7*		*Saccharimonas*	white meat	2.93	4.66E-04
OTU198	*Actinobacteria*	*Coriobacteriaceae*	*uncultured*	white meat	2.92	8.45E-08
OTU569	*Firmicutes*	*Erysipelotrichaceae*	*norank*	white meat	2.81	0.001
OTU372	*Firmicutes*	*Eubacteriaceae*	*Eubacterium*	white meat	2.94	1.66E-07
OTU266	*Firmicutes*	*Family_XIII*	*Incertae_Sedis*	white meat	3.11	5.34E-06

OTU200	*Firmicutes*	*Family_XIII*	*uncultured*	white meat	3.07	2.15E-04
OTU422	*Firmicutes*	*Family_XIII*	*uncultured*	white meat	2.69	0.001
OTU247	*Firmicutes*	*Erysipelotrichaceae*	*Incertae_Sedis*	white meat	3.58	5.72E-05
OTU149	*Firmicutes*	*Lactobacillaceae*	*Lactobacillus*	white meat	3.55	2.33E-05
OTU437	*Firmicutes*	*Lactobacillaceae*	*Lactobacillus*	white meat	3.75	3.57E-05
OTU522	*Firmicutes*	*Lactobacillaceae*	*Lactobacillus*	white meat	4.40	2.60E-05
OTU60	*Firmicutes*	*Lactobacillaceae*	*Lactobacillus*	white meat	4.34	4.51E-05
OTU817	*Firmicutes*	*Lactobacillaceae*	*Lactobacillus*	white meat	4.89	5.10E-04
OTU350	*Bacteroidetes*	*Rikenellaceae*	*RC9_gut_group*	white meat	3.04	4.35E-04
OTU794	*Bacteroidetes*	*Rikenellaceae*	*RC9_gut_group*	white meat	2.72	0.014
OTU225	*Firmicutes*	*Ruminococcaceae*	*uncultured*	white meat	2.68	0.001
OTU228	*Firmicutes*	*Ruminococcaceae*	*uncultured*	white meat	3.28	2.79E-04
OTU571	*Actinobacteria*	*Coriobacteriaceae*	*Slackia*	white meat	3.09	3.35E-07
OTU696	*Firmicutes*	*Staphylococcaceae*	*Staphylococcus*	white meat	3.66	3.25E-05
OTU140	*Firmicutes*	*Streptococcaceae*	*Streptococcus*	white meat	2.75	8.15E-06
OTU526	*Firmicutes*	*Streptococcaceae*	*Streptococcus*	white meat	3.06	1.86E-05

Supplementary Table S4 The differentially caecal bacterial communities between red meat and white meat class using LEfSe at the OTU level.

OTU ID	Toxon			Riched class	LDA Score (log10)	p value
	Phylum	Family	Genus			
OTU768	*Bacteroidetes*	*Rikenellaceae*	*Alistipes*	red meat	2.46	0.042
OTU639	*Firmicutes*	*Erysipelotrichaceae*	*Allobaculum*	red meat	3.57	0.038
OTU722	*Bacteroidetes*	*Prevotellaceae*	*Alloprevotella*	red meat	3.40	0.010
OTU673	*Firmicutes*	*Ruminococcaceae*	*Anaerotruncus*	red meat	3.63	0.029
OTU342	*Firmicutes*	*Family_XIII*	*Anaerovorax*	red meat	2.66	0.020
OTU764	*Bacteria_unclassed*			red meat	2.84	0.007
OTU560	*Bacteroidetes*	*Bacteroidaceae*	*Bacteroides*	red meat	3.81	3.43E-04
OTU283	*Bacteroidetes*	*Bacteroidaceae*	*Bacteroides*	red meat	2.29	0.010
OTU26	*Bacteroidetes*	*Bacteroidaceae*	*Bacteroides*	red meat	3.24	0.016
OTU414	*Bacteroidetes*	*Bacteroidaceae*	*Bacteroides*	red meat	3.05	0.025
OTU3	*Firmicutes*	*Lachnospiraceae*	*Blautia*	red meat	3.10	0.019
OTU502	*Firmicutes*	*Lachnospiraceae*	*Blautia*	red meat	2.22	0.048
OTU49	*Bacteroidetes*	*Porphyromonadaceae*	*Butyricimonas*	red meat	2.66	7.36E-05
OTU254	*Bacteroidetes*	*Porphyromonadaceae*	*Butyricimonas*	red meat	2.62	0.021
OTU146	*TM7*			red meat	2.41	0.047
OTU620	*Firmicutes*	*Christensenellaceae*	*uncultured*	red meat	2.52	0.017
OTU69	*Firmicutes*	*Christensenellaceae*	*uncultured*	red meat	2.01	0.020
OTU552	*Firmicutes*	*Lachnospiraceae*	*Coprococcus*	red meat	2.39	0.003
OTU643	*Firmicutes*	*Defluviitaleaceae*	*uncultured*	red meat	3.27	0.007
OTU130	*Proteobacteria*	*Desulfovibrionaceae*	*Desulfovibrio*	red meat	2.79	0.002
OTU699	*Firmicutes*	*Erysipelotrichaceae*	*uncultured*	red meat	2.35	0.016
OTU244	*Firmicutes*	*Family_XIII*	*uncultured*	red meat	2.33	0.001
OTU429	*Firmicutes*	*Family_XIII*	*uncultured*	red meat	2.88	0.009
OTU109	*Firmicutes*	*Family_XIII*	*uncultured*	red meat	2.42	0.014
OTU729	*Firmicutes*	*Ruminococcaceae*	*Intestinimonas*	red meat	3.07	0.019
OTU320	*Firmicutes*	*Lachnospiraceae*	*Incertae_Sedis*	red meat	2.29	0.024
OTU596	*Firmicutes*	*Lachnospiraceae*	*uncultured*	red meat	2.28	0.024
OTU136	*Firmicutes*	*Lachnospiraceae*	*uncultured*	red meat	2.44	0.012
OTU191	*Firmicutes*	*Lachnospiraceae*	*uncultured*	red meat	2.30	0.014
OTU240	*Firmicutes*	*Lachnospiraceae*	*uncultured*	red meat	2.39	0.014
OTU698	*Firmicutes*	*Lachnospiraceae*	*uncultured*	red meat	2.33	0.034
OTU276	*Firmicutes*	*Ruminococcaceae*	*Oscillibacter*	red meat	2.92	9.11E-04
OTU134	*Firmicutes*	*Ruminococcaceae*	*Oscillibacter*	red meat	3.04	0.001
OTU491	*Firmicutes*	*Ruminococcaceae*	*Oscillibacter*	red meat	2.39	0.002
OTU726	*Firmicutes*	*Ruminococcaceae*	*Oscillibacter*	red meat	2.11	0.003
OTU343	*Bacteroidetes*	*p-2534-18B5_gut_group*	*norank*	red meat	3.85	0.004
OTU239	*Bacteroidetes*	*p-2534-18B5_gut_group*	*norank*	red meat	3.30	0.007
OTU767	*Bacteroidetes*	*Porphyromonadaceae*	*Parabacteroides*	red meat	2.57	0.007
OTU507	*Firmicutes*	*Peptococcaceae*	*uncultured*	red meat	2.78	3.96E-04
OTU488	*Firmicutes*	*Peptococcaceae*	*Peptococcus*	red meat	2.14	0.001

OTU304	*Firmicutes*	*Peptococcaceae*	*Peptococcus*	red meat	2.33	0.002
OTU774	*Firmicutes*	*Peptococcaceae*	*Peptococcus*	red meat	2.18	0.003
OTU580	*Firmicutes*	*Peptococcaceae*	*Peptococcus*	red meat	2.96	0.031
OTU337	*Bacteroidetes*	*Prevotellaceae*	*uncultured*	red meat	2.22	0.005
OTU65	*Bacteroidetes*	*Prevotellaceae*	*uncultured*	red meat	3.64	0.030
OTU675	*Bacteroidetes*	*Prevotellaceae*	*uncultured*	red meat	2.18	0.032
OTU536	*Firmicutes*	*Veillonellaceae*	*Quinella*	red meat	3.62	0.026
OTU355	*Bacteroidetes*	*Rikenellaceae*	*RC9_gut_group*	red meat	3.34	0.007
OTU87	*Bacteroidetes*	*Rikenellaceae*	*RC9_gut_group*	red meat	2.44	0.009
OTU485	*Tenericutes*	*norank*	*RF9_norank*	red meat	2.58	4.26E-04
OTU260	*Tenericutes*	*norank*	*RF9_norank*	red meat	2.46	0.016
OTU185	*Firmicutes*	*Lachnospiraceae*	*Roseburia*	red meat	2.45	0.030
OTU251	*Firmicutes*	*Ruminococcaceae*	*Incertae_Sedis*	red meat	2.90	1.88E-04
OTU545	*Firmicutes*	*Ruminococcaceae*	*Incertae_Sedis*	red meat	2.18	0.013
OTU349	*Firmicutes*	*Ruminococcaceae*	*Incertae_Sedis*	red meat	2.24	0.021
OTU629	*Firmicutes*	*Ruminococcaceae*	*uncultured*	red meat	2.42	0.004
OTU30	*Firmicutes*	*Ruminococcaceae*	*uncultured*	red meat	2.42	0.005
OTU471	*Firmicutes*	*Ruminococcaceae*	*uncultured*	red meat	2.29	0.009
OTU458	*Firmicutes*	*Ruminococcaceae*	*uncultured*	red meat	2.37	0.011
OTU576	*Firmicutes*	*Ruminococcaceae*	*uncultured*	red meat	3.17	0.021
OTU188	*Firmicutes*	*Ruminococcaceae*	*uncultured*	red meat	2.87	0.027
OTU358	*Firmicutes*	*Ruminococcaceae*	*uncultured*	red meat	2.28	0.048
OTU199	*Firmicutes*	*Ruminococcaceae*	*uncultured*	red meat	2.99	2.97E-04
OTU717	*Firmicutes*	*Ruminococcaceae*	*uncultured*	red meat	2.47	0.007
OTU754	*Firmicutes*	*Ruminococcaceae*	*uncultured*	red meat	2.31	0.010
OTU137	*Firmicutes*	*Ruminococcaceae*	*uncultured*	red meat	2.60	0.024
OTU369	*Firmicutes*	*Ruminococcaceae*	*uncultured*	red meat	2.27	0.050
OTU797	*Firmicutes*	*Ruminococcaceae*	*Ruminococcus*	red meat	3.09	0.003
OTU478	*Firmicutes*	*Ruminococcaceae*	*Ruminococcus*	red meat	3.15	0.007
OTU144	*Bacteroidetes*	*S24-7*	*norank*	red meat	4.17	0.010
OTU584	*Bacteroidetes*	*S24-7*	*norank*	red meat	2.56	2.45E-04
OTU366	*Bacteroidetes*	*S24-7*	*norank*	red meat	3.23	2.94E-04
OTU704	*Bacteroidetes*	*S24-7*	*norank*	red meat	2.46	0.001
OTU233	*Bacteroidetes*	*S24-7*	*norank*	red meat	2.67	0.002
OTU114	*Bacteroidetes*	*S24-7*	*norank*	red meat	2.19	0.002
OTU533	*Bacteroidetes*	*S24-7*	*norank*	red meat	2.25	0.004
OTU259	*Bacteroidetes*	*S24-7*	*norank*	red meat	2.41	0.012
OTU416	*Bacteroidetes*	*S24-7*	*norank*	red meat	2.45	0.026
OTU120	*Bacteroidetes*	*S24-7*	*norank*	red meat	2.69	0.030
OTU279	*Bacteroidetes*	*S24-7*	*norank*	red meat	2.87	0.032
OTU209	*Bacteroidetes*	*S24-7*	*norank*	red meat	2.07	0.042
OTU663	*Bacteroidetes*	*S24-7*	*norank*	red meat	2.63	0.050
OTU356	*Firmicutes*	*Lachnospiraceae*	*Blautia*	white meat	3.48	0.009
OTU684	*Firmicutes*	*Lachnospiraceae*	*Blautia*	white meat	2.30	0.015

OTU302	*Firmicutes*	*Christensenellaceae*	*uncultured*	white meat	2.53	0.027
OTU24	*Proteobacteria*	*Comamonadaceae*	*uncultured*	white meat	2.96	0.044
OTU198	*Actinobacteria*	*Coriobacteriaceae*	*uncultured*	white meat	2.50	3.36E-05
OTU157	*Firmicutes*	*Erysipelotrichaceae*	*uncultured*	white meat	2.63	0.006
OTU695	*Firmicutes*	*Erysipelotrichaceae*	*uncultured*	white meat	3.47	0.033
OTU266	*Firmicutes*	*Family_XIII*	*Incertae_Sedis*	white meat	2.87	0.004
OTU357	*Firmicutes*	*Lachnospiraceae*	*uncultured*	white meat	3.02	0.006
OTU585	*Firmicutes*	*Lachnospiraceae*	*uncultured*	white meat	3.20	0.008
OTU652	*Firmicutes*	*Lachnospiraceae*	*uncultured*	white meat	3.02	0.026
OTU487	*Firmicutes*	*Lachnospiraceae*	*uncultured*	white meat	2.71	0.029
OTU817	*Firmicutes*	*Lactobacillaceae*	*Lactobacillus*	white meat	5.10	1.41E-06
OTU149	*Firmicutes*	*Lactobacillaceae*	*Lactobacillus*	white meat	3.69	2.03E-06
OTU437	*Firmicutes*	*Lactobacillaceae*	*Lactobacillus*	white meat	3.75	4.44E-05
OTU60	*Firmicutes*	*Lactobacillaceae*	*Lactobacillus*	white meat	4.46	2.97E-04
OTU693	*Firmicutes*	*Lactobacillaceae*	*Lactobacillus*	white meat	3.65	0.002
OTU522	*Firmicutes*	*Lactobacillaceae*	*Lactobacillus*	white meat	4.30	0.003
OTU421	*Firmicutes*	*Lachnospiraceae*	*Marvinbryantia*	white meat	2.88	8.85E-04
OTU468	*Firmicutes*	*Lachnospiraceae*	*Marvinbryantia*	white meat	2.62	0.005
OTU359	*Tenericutes*	*norank*	*norank*	white meat	2.09	0.035
OTU228	*Firmicutes*	*Ruminococcaceae*	*uncultured*	white meat	2.77	0.035

Supplementary Table S5 Significantly different OTUs between casein and beef protein group using Metastats analysis.

	Toxon			Mean		
OTU ID	Phylum	Family	Genus	Casein	Beef	p value
OTU820	*Bacteroidetes*	*Bacteroidaceae*	*Bacteroides*	1.21%	0.06%	<0.001
OTU162	*Firmicutes*	*Ruminococcaceae*	*uncultured*	0.62%	0.02%	<0.001
OTU613	*Bacteroidetes*	*Prevotellaceae*	*uncultured*	0.26%	0.00%	<0.001
OTU657	*Firmicutes*	*Ruminococcaceae*	*uncultured*	0.25%	0.00%	<0.001
OTU66	*Bacteroidetes*	*Prevotellaceae*	*uncultured*	0.15%	0.00%	<0.001
OTU685	*Firmicutes*	*Ruminococcaceae*	*Incertae_Sedis*	0.14%	0.00%	<0.001
OTU727	*Firmicutes*	*Lachnospiraceae*	*Blautia*	0.13%	0.00%	<0.001
OTU401	*Firmicutes*	*Lactobacillaceae*	*Lactobacillus*	0.12%	0.00%	<0.001
OTU406	*Firmicutes*	*Ruminococcaceae*	*uncultured*	0.10%	0.00%	<0.001
OTU104	*Firmicutes*	*Erysipelotrichaceae*	*Allobaculum*	0.09%	0.96%	<0.001
OTU639	*Firmicutes*	*Erysipelotrichaceae*	*Allobaculum*	0.00%	1.42%	<0.001
OTU571	*Actinobacteria*	*Coriobacteriaceae*	*Slackia*	0.00%	0.25%	<0.001
OTU489	*Firmicutes*	*Erysipelotrichaceae*	*uncultured*	0.00%	0.29%	<0.001
OTU522	*Firmicutes*	*Lactobacillaceae*	*Lactobacillus*	0.61%	5.28%	<0.01
OTU779	*Firmicutes*	*Lachnospiraceae*	*Incertae_Sedis*	0.42%	0.04%	<0.01
OTU227	*Firmicutes*	*Lachnospiraceae*	*Roseburia*	5.05%	0.42%	<0.01
OTU757	*Firmicutes*	*Streptococcaceae*	*Streptococcus*	0.39%	0.07%	<0.01
OTU717	*Firmicutes*	*Ruminococcaceae*		0.25%	0.05%	<0.01
OTU405	*Firmicutes*	*Lactobacillaceae*	*Lactobacillus*	0.10%	0.02%	<0.01
OTU457	*Firmicutes*	*Family_XIII*	*Mogibacterium*	0.03%	0.13%	<0.01
OTU429	*Firmicutes*	*Family_XIII*	*uncultured*	0.02%	0.16%	<0.01
OTU173	*Firmicutes*	*Lachnospiraceae*		0.00%	0.10%	<0.01
OTU585	*Firmicutes*	*Lachnospiraceae*		1.83%	0.26%	<0.01
OTU247	*Firmicutes*	*Erysipelotrichaceae*	*Incertae_Sedis*	0.14%	0.93%	<0.01
OTU576	*Firmicutes*	*Ruminococcaceae*	*uncultured*	0.07%	0.48%	<0.01
OTU526	*Firmicutes*	*Streptococcaceae*	*Streptococcus*	0.14%	0.38%	<0.01
OTU736	*Firmicutes*	*Erysipelotrichaceae*	*uncultured*	0.07%	0.19%	<0.01
OTU423	*Firmicutes*	*Lachnospiraceae*	*Blautia*	1.83%	0.00%	<0.05
OTU590	*Firmicutes*	*Peptostreptococcaceae*	*Incertae_Sedis*	7.25%	3.47%	<0.05
OTU68	*Firmicutes*	*Erysipelotrichaceae*	*uncultured*	0.01%	0.12%	<0.05
OTU327	*Proteobacteria*	*Pasteurellaceae*	*Pasteurella*	0.04%	0.38%	<0.05
OTU313	*Tenericutes*	*norank*	*norank*	0.18%	0.96%	<0.05
OTU544	*Firmicutes*	*Ruminococcaceae*		0.05%	0.11%	<0.05
OTU434	*Bacteroidetes*	*Prevotellaceae*	*Alloprevotella*	1.84%	0.36%	<0.05
OTU123	*Firmicutes*	*Ruminococcaceae*	*uncultured*	2.87%	1.46%	<0.05
OTU197	*Firmicutes*	*Ruminococcaceae*	*uncultured*	0.41%	0.96%	<0.05
OTU89	*Firmicutes*	*Family_XI*	*Gemella*	0.17%	0.43%	<0.05
OTU355	*Bacteroidetes*	*Rikenellaceae*	*RC9_gut_group*	0.04%	0.55%	<0.05
OTU695	*Firmicutes*	*Erysipelotrichaceae*	*uncultured*	1.07%	0.28%	<0.05
OTU259	*Bacteroidetes*	*S24-7*	*norank*	0.01%	0.10%	<0.05

Supplementary Table S6 Significantly different OTUs between casein and chicken protein group using Metastats analysis.

OTU ID	Toxon			mean		
	Phylum	Family	Genus	casein	chicken	p value
OTU748	*Bacteroidetes*	*S24-7*	*norank*	3.02%	0.26%	<0.001
OTU560	*Bacteroidetes*	*Bacteroidaceae*	*Bacteroides*	1.97%	0.13%	<0.001
OTU162	*Firmicutes*	*Ruminococcaceae*	*uncultured*	0.62%	0.03%	<0.001
OTU522	*Firmicutes*	*Lactobacillaceae*	*Lactobacillus*	0.61%	8.72%	<0.001
OTU757	*Firmicutes*	*Streptococcaceae*	*Streptococcus*	0.39%	0.05%	<0.001
OTU613	*Bacteroidetes*	*Prevotellaceae*	*uncultured*	0.26%	0.00%	<0.001
OTU717	*Firmicutes*	*Ruminococcaceae*		0.25%	0.02%	<0.001
OTU657	*Firmicutes*	*Ruminococcaceae*	*uncultured*	0.25%	0.00%	<0.001
OTU66	*Bacteroidetes*	*Prevotellaceae*	*uncultured*	0.15%	0.00%	<0.001
OTU685	*Firmicutes*	*Ruminococcaceae*	*Incertae_Sedis*	0.14%	0.00%	<0.001
OTU727	*Firmicutes*	*Lachnospiraceae*	*Blautia*	0.13%	0.00%	<0.001
OTU383	*Tenericutes*	*norank*	*norank*	0.12%	0.00%	<0.001
OTU401	*Firmicutes*	*Lactobacillaceae*	*Lactobacillus*	0.12%	0.00%	<0.001
OTU406	*Firmicutes*	*Ruminococcaceae*	*uncultured*	0.10%	0.00%	<0.001
OTU149	*Firmicutes*	*Lactobacillaceae*	*Lactobacillus*	0.09%	1.13%	<0.001
OTU104	*Firmicutes*	*Erysipelotrichaceae*	*Allobaculum*	0.09%	0.81%	<0.001
OTU266	*Firmicutes*	*Family_XIII*	*Incertae_Sedis*	0.04%	0.39%	<0.001
OTU639	*Firmicutes*	*Erysipelotrichaceae*	*Allobaculum*	0.00%	0.47%	<0.001
OTU571	*Actinobacteria*	*Coriobacteriaceae*	*Slackia*	0.00%	0.35%	<0.001
OTU393	*Firmicutes*	*Erysipelotrichaceae*	*Allobaculum*	1.06%	0.01%	<0.01
OTU416	*Bacteroidetes*	*S24-7*	*norank*	0.24%	0.03%	<0.01
OTU696	*Firmicutes*	*Staphylococcaceae*	*Staphylococcus*	0.17%	0.76%	<0.01
OTU693	*Firmicutes*	*Lactobacillaceae*	*Lactobacillus*	0.15%	2.39%	<0.01
OTU405	*Firmicutes*	*Lactobacillaceae*	*Lactobacillus*	0.10%	0.02%	<0.01
OTU321	*Firmicutes*	*Ruminococcaceae*	*uncultured*	0.02%	0.33%	<0.01
OTU434	*Bacteroidetes*	*Prevotellaceae*	*Alloprevotella*	1.84%	0.08%	<0.01
OTU797	*Firmicutes*	*Ruminococcaceae*	*Ruminococcus*	0.35%	0.02%	<0.01
OTU233	*Bacteroidetes*	*S24-7*	*norank*	0.17%	0.04%	<0.01
OTU526	*Firmicutes*	*Streptococcaceae*	*Streptococcus*	0.14%	0.36%	<0.01
OTU478	*Firmicutes*	*Ruminococcaceae*	*Ruminococcus*	0.43%	0.05%	<0.01
OTU247	*Firmicutes*	*Erysipelotrichaceae*	*Incertae_Sedis*	0.14%	1.17%	<0.01
OTU63	*Bacteroidetes*	*Prevotellaceae*	*uncultured*	0.13%	0.00%	<0.01
OTU817	*Firmicutes*	*Lactobacillaceae*	*Lactobacillus*	6.85%	26.47%	<0.01
OTU134	*Firmicutes*	*Ruminococcaceae*	*Oscillibacter*	0.22%	0.03%	<0.01
OTU700	*Firmicutes*	*Ruminococcaceae*	*uncultured*	0.17%	0.00%	<0.01
OTU327	*Proteobacteria*	*Pasteurellaceae*	*Pasteurella*	0.04%	0.18%	<0.01
OTU276	*Firmicutes*	*Ruminococcaceae*	*Oscillibacter*	0.31%	0.08%	<0.05
OTU60	*Firmicutes*	*Lactobacillaceae*	*Lactobacillus*	1.10%	6.46%	<0.05
OTU213	*Bacteroidetes*	*S24-7*	*norank*	0.84%	0.04%	<0.05
OTU229	*Bacteroidetes*	*S24-7*	*norank*	0.13%	0.00%	<0.05

OTU429	*Firmicutes*	*Family_XIII*	*uncultured*	0.02%	0.23%	<0.05
OTU820	*Bacteroidetes*	*Bacteroidaceae*	*Bacteroides*	1.21%	0.11%	<0.05
OTU643	*Firmicutes*	*Defluviitaleaceae*	*uncultured*	0.89%	0.28%	<0.05
OTU26	*Bacteroidetes*	*Bacteroidaceae*	*Bacteroides*	0.29%	0.05%	<0.05
OTU555	*Firmicutes*	*Ruminococcaceae*	*Intestinimonas*	0.08%	0.22%	<0.05
OTU722	*Bacteroidetes*	*Prevotellaceae*	*Alloprevotella*	0.87%	0.08%	<0.05
OTU366	*Bacteroidetes*	*S24-7*	*norank*	0.20%	0.07%	<0.05
OTU536	*Firmicutes*	*Veillonellaceae*	*Quinella*	1.65%	0.42%	<0.05
OTU425	*Firmicutes*	*Ruminococcaceae*	*uncultured*	0.12%	0.00%	<0.05
OTU121	*Firmicutes*	*Ruminococcaceae*		0.10%	0.02%	<0.05
OTU736	*Firmicutes*	*Erysipelotrichaceae*	*uncultured*	0.07%	0.17%	<0.05
OTU821	*Firmicutes*	*Streptococcaceae*	*Lactococcus*	0.56%	0.23%	<0.05
OTU89	*Firmicutes*	*Family_XI*	*Gemella*	0.17%	0.40%	<0.05
OTU19	*Bacteroidetes*	*Porphyromonadaceae*	*Parabacteroides*	0.15%	0.04%	<0.05
OTU225	*Firmicutes*	*Ruminococcaceae*	*uncultured*	0.01%	0.12%	<0.05

Supplementary Table S7 Significantly different OTUs between casein and fish protein group using Metastats analysis.

	Toxon			mean		p value
OTU ID	Phylum	Family	Genus	Casein	Fish	
OTU162	*Firmicutes*	*Ruminococcaceae*	*uncultured*	0.62%	0.05%	<0.001
OTU522	*Firmicutes*	*Lactobacillaceae*	*Lactobacillus*	0.61%	4.35%	<0.001
OTU757	*Firmicutes*	*Streptococcaceae*	*Streptococcus*	0.39%	0.04%	<0.001
OTU276	*Firmicutes*	*Ruminococcaceae*	*Oscillibacter*	0.31%	0.05%	<0.001
OTU613	*Bacteroidetes*	*Prevotellaceae*	*uncultured*	0.26%	0.01%	<0.001
OTU717	*Firmicutes*	*Ruminococcaceae*		0.25%	0.01%	<0.001
OTU251	*Firmicutes*	*Ruminococcaceae*	*Incertae_Sedis*	0.21%	0.01%	<0.001
OTU366	*Bacteroidetes*	*S24-7*	*norank*	0.20%	0.01%	<0.001
OTU104	*Firmicutes*	*Erysipelotrichaceae*	*Allobaculum*	0.09%	0.41%	<0.001
OTU639	*Firmicutes*	*Erysipelotrichaceae*	*Allobaculum*	0.00%	1.08%	<0.001
OTU696	*Firmicutes*	*Staphylococcaceae*	*Staphylococcus*	0.17%	1.61%	<0.01
OTU233	*Bacteroidetes*	*S24-7*	*norank*	0.17%	0.03%	<0.01
OTU526	*Firmicutes*	*Streptococcaceae*	*Streptococcus*	0.14%	0.30%	<0.01
OTU406	*Firmicutes*	*Ruminococcaceae*	*uncultured*	0.10%	0.01%	<0.01
OTU416	*Bacteroidetes*	*S24-7*	*norank*	0.24%	0.03%	<0.01
OTU149	*Firmicutes*	*Lactobacillaceae*	*Lactobacillus*	0.09%	0.65%	<0.01
OTU748	*Bacteroidetes*	*S24-7*	*norank*	3.02%	0.41%	<0.01
OTU560	*Bacteroidetes*	*Bacteroidaceae*	*Bacteroides*	1.97%	0.30%	<0.01
OTU300	*Firmicutes*	*Ruminococcaceae*	*uncultured*	1.26%	4.53%	<0.01
OTU134	*Firmicutes*	*Ruminococcaceae*	*Oscillibacter*	0.22%	0.03%	<0.01
OTU266	*Firmicutes*	*Family_XIII*	*Incertae_Sedis*	0.04%	0.14%	<0.01
OTU393	*Firmicutes*	*Erysipelotrichaceae*	*Allobaculum*	1.06%	0.02%	<0.01
OTU817	*Firmicutes*	*Lactobacillaceae*	*Lactobacillus*	6.85%	24.46%	<0.01
OTU673	*Firmicutes*	*Ruminococcaceae*	*Anaerotruncus*	1.61%	0.66%	<0.01
OTU693	*Firmicutes*	*Lactobacillaceae*	*Lactobacillus*	0.15%	0.60%	<0.01
OTU227	*Firmicutes*	*Lachnospiraceae*	*Roseburia*	5.05%	0.56%	<0.05
OTU123	*Firmicutes*	*Ruminococcaceae*	*uncultured*	2.87%	1.49%	<0.05
OTU295	*Firmicutes*	*Ruminococcaceae*	*uncultured*	0.13%	0.02%	<0.05
OTU199	*Firmicutes*	*Ruminococcaceae*		0.20%	0.04%	<0.05
OTU120	*Bacteroidetes*	*S24-7*	*norank*	0.18%	0.05%	<0.05
OTU60	*Firmicutes*	*Lactobacillaceae*	*Lactobacillus*	1.10%	4.64%	<0.05
OTU797	*Firmicutes*	*Ruminococcaceae*	*Ruminococcus*	0.35%	0.06%	<0.05
OTU765	*Firmicutes*	*Ruminococcaceae*	*uncultured*	1.00%	3.03%	<0.05
OTU434	*Bacteroidetes*	*Prevotellaceae*	*Alloprevotella*	1.84%	0.30%	<0.05
OTU213	*Bacteroidetes*	*S24-7*	*norank*	0.84%	0.06%	<0.05
OTU478	*Firmicutes*	*Ruminococcaceae*	*Ruminococcus*	0.43%	0.12%	<0.05
OTU821	*Firmicutes*	*Streptococcaceae*	*Lactococcus*	0.56%	0.21%	<0.05
OTU536	*Firmicutes*	*Veillonellaceae*	*Quinella*	1.65%	0.42%	<0.05
OTU3	*Firmicutes*	*Lachnospiraceae*	*Blautia*	0.54%	0.02%	<0.05

Supplementary Table S8 Significantly different OTUs between casein and pork protein group using Metastats analysis

OTU ID	Toxon			mean		p value
	Phylum	Family	Genus	Casein	Pork	
OTU162	*Firmicutes*	*Ruminococcaceae*	*uncultured*	0.62%	0.02%	<0.001
OTU613	*Bacteroidetes*	*Prevotellaceae*	*uncultured*	0.26%	0.01%	<0.001
OTU717	*Firmicutes*	*Ruminococcaceae*		0.25%	0.02%	<0.001
OTU66	*Bacteroidetes*	*Prevotellaceae*	*uncultured*	0.15%	0.00%	<0.001
OTU685	*Firmicutes*	*Ruminococcaceae*	*Incertae_Sedis*	0.14%	0.00%	<0.001
OTU63	*Bacteroidetes*	*Prevotellaceae*	*uncultured*	0.13%	0.00%	<0.001
OTU401	*Firmicutes*	*Lactobacillaceae*	*Lactobacillus*	0.12%	0.00%	<0.001
OTU406	*Firmicutes*	*Ruminococcaceae*	*uncultured*	0.10%	0.00%	<0.001
OTU429	*Firmicutes*	*Family_XIII*	*uncultured*	0.02%	0.25%	<0.001
OTU639	*Firmicutes*	*Erysipelotrichaceae*	*Allobaculum*	0.00%	0.93%	<0.001
OTU571	*Actinobacteria*	*Coriobacteriaceae*	*Slackia*	0.00%	0.24%	<0.001
OTU590	*Firmicutes*	*Peptostreptococcaceae*	*Incertae_Sedis*	7.25%	1.88%	<0.01
OTU695	*Firmicutes*	*Erysipelotrichaceae*	*uncultured*	1.07%	0.11%	<0.01
OTU393	*Firmicutes*	*Erysipelotrichaceae*	*Allobaculum*	1.06%	0.01%	<0.01
OTU603	*Firmicutes*	*Lachnospiraceae*	*Incertae_Sedis*	0.33%	0.01%	<0.01
OTU696	*Firmicutes*	*Staphylococcaceae*	*Staphylococcus*	0.17%	1.67%	<0.01
OTU350	*Bacteroidetes*	*Rikenellaceae*	*RC9_gut_group*	0.00%	0.14%	<0.01
OTU757	*Firmicutes*	*Streptococcaceae*	*Streptococcus*	0.39%	0.06%	<0.01
OTU247	*Firmicutes*	*Erysipelotrichaceae*	*Incertae_Sedis*	0.14%	1.53%	<0.01
OTU68	*Firmicutes*	*Erysipelotrichaceae*	*uncultured*	0.01%	0.20%	<0.01
OTU434	*Bacteroidetes*	*Prevotellaceae*	*Alloprevotella*	1.84%	0.09%	<0.01
OTU779	*Firmicutes*	*Lachnospiraceae*	*Incertae_Sedis*	0.42%	0.03%	<0.01
OTU123	*Firmicutes*	*Ruminococcaceae*	*uncultured*	2.87%	1.11%	<0.01
OTU416	*Bacteroidetes*	*S24-7*	*norank*	0.24%	0.03%	<0.01
OTU585	*Firmicutes*	*Lachnospiraceae*		1.83%	0.25%	<0.01
OTU295	*Firmicutes*	*Ruminococcaceae*	*uncultured*	0.13%	0.02%	<0.01
OTU357	*Firmicutes*	*Lachnospiraceae*		0.64%	0.13%	<0.01
OTU331	*Bacteroidetes*	*Prevotellaceae*	*uncultured*	0.00%	0.16%	<0.05
OTU258	*Firmicutes*	*Lachnospiraceae*	*Marvinbryantia*	0.10%	0.00%	<0.05
OTU197	*Firmicutes*	*Ruminococcaceae*	*uncultured*	0.41%	1.04%	<0.05
OTU233	*Bacteroidetes*	*S24-7*	*norank*	0.17%	0.06%	<0.05
OTU298	*Firmicutes*	*Ruminococcaceae*	*Ruminococcus*	0.00%	0.22%	<0.05
OTU820	*Bacteroidetes*	*Bacteroidaceae*	*Bacteroides*	1.21%	0.13%	<0.05
OTU188	*Firmicutes*	*Ruminococcaceae*	*uncultured*	0.02%	0.23%	<0.05
OTU653	*Firmicutes*	*Family_XIII*	*Incertae_Sedis*	0.36%	0.14%	<0.05
OTU576	*Firmicutes*	*Ruminococcaceae*	*uncultured*	0.07%	0.26%	<0.05
OTU121	*Firmicutes*	*Ruminococcaceae*		0.10%	0.02%	<0.05
OTU748	*Bacteroidetes*	*S24-7*	*norank*	3.02%	0.72%	<0.05
OTU225	*Firmicutes*	*Ruminococcaceae*	*uncultured*	0.01%	0.17%	<0.05
OTU300	*Firmicutes*	*Ruminococcaceae*	*uncultured*	1.26%	7.87%	<0.05

OTU560	*Bacteroidetes*	*Bacteroidaceae*	*Bacteroides*	1.97%	0.52%	<0.05
OTU313	*Tenericutes*	*norank*	*norank*	0.18%	0.61%	<0.05
OTU332	*Firmicutes*	*Family_XIII*	*Incertae_Sedis*	0.11%	0.06%	<0.05
OTU457	*Firmicutes*	*Family_XIII*	*Mogibacterium*	0.03%	0.10%	<0.05
OTU467	*Firmicutes*	*Ruminococcaceae*	*Faecalibacterium*	2.20%	0.33%	<0.05
OTU765	*Firmicutes*	*Ruminococcaceae*	*uncultured*	1.00%	7.09%	<0.05
OTU355	*Bacteroidetes*	*Rikenellaceae*	*RC9_gut_group*	0.04%	0.30%	<0.05
OTU288	*Tenericutes*	*norank*	*norank*	1.22%	2.43%	<0.05
OTU161	*Bacteroidetes*	*Prevotellaceae*	*uncultured*	0.51%	1.94%	<0.05
OTU104	*Firmicutes*	*Erysipelotrichaceae*	*Allobaculum*	0.09%	0.30%	<0.05

Supplementary Table S9 Significantly different OTUs between casein and soy protein group using Metastats analysis

OTU ID	Toxon			mean		p value
	Phylum	Family	Genus	casein	soy	
OTU123	*Firmicutes*	*Ruminococcaceae*	*uncultured*	2.87%	0.58%	<0.001
OTU357	*Firmicutes*	*Lachnospiraceae*		0.64%	0.11%	<0.001
OTU779	*Firmicutes*	*Lachnospiraceae*	*Incertae_Sedis*	0.42%	0.01%	<0.001
OTU757	*Firmicutes*	*Streptococcaceae*	*Streptococcus*	0.39%	0.02%	<0.001
OTU437	*Firmicutes*	*Lactobacillaceae*	*Lactobacillus*	0.35%	0.01%	<0.001
OTU717	*Firmicutes*	*Ruminococcaceae*		0.25%	0.02%	<0.001
OTU66	*Bacteroidetes*	*Prevotellaceae*	*uncultured*	0.15%	1.19%	<0.001
OTU405	*Firmicutes*	*Lactobacillaceae*	*Lactobacillus*	0.10%	0.00%	<0.001
OTU639	*Firmicutes*	*Erysipelotrichaceae*	*Allobaculum*	0.00%	2.25%	<0.001
OTU521	*Bacteroidetes*	*S24-7*	*norank*	0.00%	0.25%	<0.001
OTU223	*Firmicutes*	*Lachnospiraceae*		0.00%	0.14%	<0.001
OTU821	*Firmicutes*	*Streptococcaceae*	*Lactococcus*	0.56%	0.10%	<0.01
OTU323	*Actinobacteria*	*Coriobacteriaceae*	*Collinsella*	0.36%	0.02%	<0.01
OTU603	*Firmicutes*	*Lachnospiraceae*	*Incertae_Sedis*	0.33%	0.01%	<0.01
OTU719	*Bacteroidetes*	*Prevotellaceae*	*Prevotella*	0.06%	0.37%	<0.01
OTU355	*Bacteroidetes*	*Rikenellaceae*	*RC9_gut_group*	0.04%	0.29%	<0.01
OTU2	*Firmicutes*	*Ruminococcaceae*	*uncultured*	0.01%	0.30%	<0.01
OTU8	*Bacteroidetes*	*S24-7*	*norank*	0.01%	0.12%	<0.01
OTU406	*Firmicutes*	*Ruminococcaceae*	*uncultured*	0.10%	0.01%	<0.01
OTU728	*Bacteroidetes*	*S24-7*	*norank*	0.03%	0.44%	<0.01
OTU68	*Firmicutes*	*Erysipelotrichaceae*	*uncultured*	0.01%	0.15%	<0.01
OTU92	*Bacteroidetes*	*Bacteroidaceae*	*Bacteroides*	0.19%	0.01%	<0.01
OTU332	*Firmicutes*	*Family_XIII*	*Incertae_Sedis*	0.11%	0.04%	<0.01
OTU716	*Firmicutes*	*Lachnospiraceae*	*uncultured*	0.37%	0.12%	<0.01
OTU289	*Firmicutes*	*Lachnospiraceae*	*Blautia*	0.02%	0.37%	<0.05
OTU820	*Bacteroidetes*	*Bacteroidaceae*	*Bacteroides*	1.21%	0.11%	<0.05
OTU65	*Bacteroidetes*	*Prevotellaceae*	*uncultured*	0.34%	1.66%	<0.05
OTU653	*Firmicutes*	*Family_XIII*	*Incertae_Sedis*	0.36%	0.12%	<0.05
OTU535	*Firmicutes*	*Lachnospiraceae*	*Blautia*	0.08%	0.31%	<0.05
OTU233	*Bacteroidetes*	*S24-7*	*norank*	0.17%	0.05%	<0.05
OTU162	*Firmicutes*	*Ruminococcaceae*	*uncultured*	0.62%	0.21%	<0.05
OTU712	*Bacteroidetes*	*S24-7*	*norank*	0.05%	0.15%	<0.05
OTU467	*Firmicutes*	*Ruminococcaceae*	*Faecalibacterium*	2.20%	0.33%	<0.05
OTU366	*Bacteroidetes*	*S24-7*	*norank*	0.20%	0.07%	<0.05

Supplementary Table S10 The differentially caecal bacterial communities between meat class and soy protein group using LEfSe at the OTU level.

OTU ID	Toxon			Riched class	LDA Score (log10)	p value
	Phylum	Family	Genus			
OTU323	*Actinobacteria*	*Coriobacteriaceae*	*Collinsella*	Meat	3.44	2.19E-06
OTU571	*Actinobacteria*	*Coriobacteriaceae*	*Slackia*	Meat	3.05	6.43E-06
OTU287	*Bacteroidetes*	*Flavobacteriaceae*	*Flavobacterium*	Meat	2.40	0.0095
OTU246	*Bacteroidetes*	*Prevotellaceae*	*Alloprevotella*	Meat	3.50	4.17E-04
OTU519	*TM7*		*Saccharimonas*	Meat	2.19	5.29E-04
OTU773	*Firmicutes*	*Bacillaceae*	*Bacillus*	Meat	2.14	0.0042
OTU723	*Firmicutes*	*Carnobacteriaceae*	*Carnobacterium*	Meat	2.18	0.0012
OTU250	*Firmicutes*	*Christensenellaceae*	*uncultured*	Meat	3.06	7.72E-04
OTU418	*Firmicutes*	*Christensenellaceae*	*uncultured*	Meat	2.77	0.0018
OTU104	*Firmicutes*	*Erysipelotrichaceae*	*Allobaculum*	Meat	3.45	8.88E-05
OTU247	*Firmicutes*	*Erysipelotrichaceae*	*Incertae_Sedis*	Meat	3.68	8.33E-06
OTU372	*Firmicutes*	*Eubacteriaceae*	*Eubacterium*	Meat	2.30	4.42E-05
OTU266	*Firmicutes*	*Family_XIII*	*Incertae_Sedis*	Meat	2.89	3.26E-04
OTU779	*Firmicutes*	*Lachnospiraceae*	*Incertae_Sedis*	Meat	2.46	5.42E-04
OTU405	*Firmicutes*	*Lactobacillaceae*	*Lactobacillus*	Meat	3.45	0.0027
OTU687	*Firmicutes*	*Ruminococcaceae*	*Anaerofilum*	Meat	2.13	0.0034
OTU555	*Firmicutes*	*Ruminococcaceae*	*Intestinimonas*	Meat	2.85	3.64E-04
OTU197	*Firmicutes*	*Ruminococcaceae*	*uncultured*	Meat	3.51	0.0016
OTU711	*Firmicutes*	*Ruminococcaceae*	*uncultured*	Meat	2.32	2.04E-05
OTU99	*Firmicutes*	*Ruminococcaceae*		Meat	2.12	0.0087
OTU696	*Firmicutes*	*Staphylococcaceae*	*Staphylococcus*	Meat	3.65	3.99E-04
OTU477	*Firmicutes*	*Streptococcaceae*	*Lactococcus*	Meat	3.74	0.0054
OTU821	*Firmicutes*	*Streptococcaceae*	*Lactococcus*	Meat	2.99	0.0054
OTU360	*Proteobacteria*	*Neisseriaceae*	*Kingella*	Meat	2.18	0.0019
OTU689	*Proteobacteria*	*Oxalobacteraceae*	*Janthinobacterium*	Meat	2.21	0.0050
OTU610	*Proteobacteria*	*Pseudomonadaceae*	*Pseudomonas*	Meat	2.90	0.0054
OTU35	*Proteobacteria*	*Pseudomonadaceae*	*Pseudomonas*	Meat	2.84	0.0092
OTU412	*Bacteroidetes*	*Bacteroidaceae*	*Bacteroides*	Soy	2.28	7.17E-08
OTU560	*Bacteroidetes*	*Bacteroidaceae*	*Bacteroides*	Soy	4.13	3.09E-04
OTU283	*Bacteroidetes*	*Bacteroidaceae*	*Bacteroides*	Soy	2.72	2.67E-04
OTU26	*Bacteroidetes*	*Bacteroidaceae*	*Bacteroides*	Soy	3.11	0.0041
OTU167	*Bacteroidetes*	*Porphyromonadaceae*	*Odoribacter*	Soy	2.40	0.0067
OTU677	*Bacteroidetes*	*Porphyromonadaceae*	*Parabacteroides*	Soy	2.59	8.60E-07
OTU434	*Bacteroidetes*	*Prevotellaceae*	*Alloprevotella*	Soy	4.19	0.0104
OTU147	*Bacteroidetes*	*Prevotellaceae*	*Prevotella*	Soy	2.49	8.55E-06
OTU719	*Bacteroidetes*	*Prevotellaceae*	*Prevotella*	Soy	3.25	5.06E-05
OTU66	*Bacteroidetes*	*Prevotellaceae*	*uncultured*	Soy	3.75	3.36E-12
OTU613	*Bacteroidetes*	*Prevotellaceae*	*uncultured*	Soy	3.03	1.64E-04
OTU100	*Bacteroidetes*	*Prevotellaceae*	*uncultured*	Soy	2.42	7.17E-08
OTU640	*Bacteroidetes*	*Prevotellaceae*	*uncultured*	Soy	2.52	1.06E-10

OTU269	*Bacteroidetes*	*Prevotellaceae*	*uncultured*	Soy	2.63	2.27E-10
OTU675	*Bacteroidetes*	*Prevotellaceae*		Soy	2.19	0.0029
OTU447	*Bacteroidetes*	*Rikenellaceae*	*Alistipes*	Soy	3.05	2.63E-04
OTU694	*Bacteroidetes*	*Rikenellaceae*	*RC9_gut_group*	Soy	2.38	2.27E-05
OTU292	*Bacteroidetes*	*S24-7*	*norank*	Soy	2.26	1.53E-06
OTU537	*Bacteroidetes*	*S24-7*	*norank*	Soy	2.19	4.79E-08
OTU76	*Bacteroidetes*	*S24-7*	*norank*	Soy	2.54	1.27E-06
OTU665	*Bacteroidetes*	*S24-7*	*norank*	Soy	2.62	0.0019
OTU521	*Bacteroidetes*	*S24-7*	*norank*	Soy	3.10	1.08E-08
OTU777	*Bacteroidetes*	*S24-7*	*norank*	Soy	2.75	4.98E-05
OTU416	*Bacteroidetes*	*S24-7*	*norank*	Soy	2.88	7.03E-04
OTU515	*Bacteroidetes*	*S24-7*	*norank*	Soy	2.51	5.43E-05
OTU747	*Bacteroidetes*	*S24-7*	*norank*	Soy	2.51	8.48E-07
OTU728	*Bacteroidetes*	*S24-7*	*norank*	Soy	3.27	1.17E-08
OTU282	*Bacteroidetes*	*S24-7*	*norank*	Soy	2.33	6.64E-08
OTU8	*Bacteroidetes*	*S24-7*	*norank*	Soy	2.73	1.30E-04
OTU257	*Bacteroidetes*			Soy	2.30	0.0023
OTU758	*Cyanobacteria*	*norank*	*norank*	Soy	2.14	0.0018
OTU119	*Cyanobacteria*	*norank*	*norank*	Soy	2.60	1.11E-06
OTU642	*Firmicutes*	*Clostridiaceae_1*	*Arthromitus*	Soy	2.22	1.44E-04
OTU91	*Firmicutes*	*Defluviitaleaceae*	*uncultured*	Soy	2.52	9.41E-06
OTU329	*Firmicutes*	*Lachnospiraceae*	*Blautia*	Soy	2.26	7.36E-07
OTU718	*Firmicutes*	*Lachnospiraceae*	*Blautia*	Soy	2.60	8.39E-08
OTU826	*Firmicutes*	*Lachnospiraceae*	*Blautia*	Soy	2.32	1.11E-06
OTU423	*Firmicutes*	*Lachnospiraceae*	*Blautia*	Soy	3.11	1.35E-07
OTU57	*Firmicutes*	*Lachnospiraceae*	*Blautia*	Soy	2.80	6.69E-09
OTU727	*Firmicutes*	*Lachnospiraceae*	*Blautia*	Soy	3.02	3.85E-09
OTU461	*Firmicutes*	*Lachnospiraceae*	*Blautia*	Soy	2.60	1.42E-04
OTU3	*Firmicutes*	*Lachnospiraceae*	*Blautia*	Soy	3.96	9.39E-05
OTU454	*Firmicutes*	*Lachnospiraceae*	*Oribacterium*	Soy	2.32	1.97E-04
OTU615	*Firmicutes*	*Lachnospiraceae*	*Roseburia*	Soy	2.27	4.23E-09
OTU70	*Firmicutes*	*Lachnospiraceae*	*Roseburia*	Soy	2.80	0.0014
OTU227	*Firmicutes*	*Lachnospiraceae*	*Roseburia*	Soy	4.58	0.0044
OTU444	*Firmicutes*	*Lachnospiraceae*	*uncultured*	Soy	2.14	8.02E-05
OTU126	*Firmicutes*	*Peptococcaceae*	*uncultured*	Soy	2.56	1.09E-05
OTU726	*Firmicutes*	*Ruminococcaceae*	*Oscillibacter*	Soy	2.47	3.57E-04
OTU486	*Firmicutes*	*Ruminococcaceae*	*Ruminococcus*	Soy	3.53	0.0026
OTU425	*Firmicutes*	*Ruminococcaceae*	*uncultured*	Soy	2.85	1.17E-04
OTU657	*Firmicutes*	*Ruminococcaceae*	*uncultured*	Soy	2.94	6.28E-09
OTU2	*Firmicutes*	*Ruminococcaceae*	*uncultured*	Soy	3.13	1.68E-08
OTU162	*Firmicutes*	*Ruminococcaceae*	*uncultured*	Soy	2.93	1.19E-04
OTU524	*Firmicutes*	*Ruminococcaceae*		Soy	2.43	5.71E-06
OTU387	*Proteobacteria*	*Alcaligenaceae*	*Sutterella*	Soy	2.27	0.0021
